# Assessment of the Evolution of Cancer Treatment Therapies

**DOI:** 10.3390/cancers3033279

**Published:** 2011-08-12

**Authors:** Manuel Arruebo, Nuria Vilaboa, Berta Sáez-Gutierrez, Julio Lambea, Alejandro Tres, Mónica Valladares, África González-Fernández

**Affiliations:** 1. Instituto de Nanociencia de Aragón (INA), Mariano Esquillor, Edif. I+D, University of Zaragoza, Zaragoza 50018, Spain; E-Mails: arruebom@unizar.es (M.A.); bsaez@unizar.es (B.S.); jjlambea@hotmail.com (J.L.); atreszar@unizar.es (A.T.); 2. CIBER de Bioingeniería, Biomateriales y Nanomedicina (CIBER-BBN), Zaragoza 50018, Spain; E-Mail: nvilaboa.hulp@salud.madrid.org (N.V.); 3. Hospital Universitario La Paz-IdiPAZ, Paseo de la Castellana 261, Madrid 28046, Spain; 4. Servicio de Oncología Médica, Hospital Clínico Universitario Lozano Blesa, Avda. San Juan Bosco 50009, Zaragoza, Spain; 5. Instituto Aragonés de Ciencias de la Salud (I+CS), Avda. Gómez Laguna, 25, Zaragoza 50009, Spain; 6. Lonza Biologics Porriño, A relva s/n, Porriño (Pontevedra) 36410, Spain; E-Mail: monica.valladares@lonza.com (M.V.); 7. Immunology Department, Biomedical Research Center (CINBIO), University of Vigo, Campus Lagoas Marcosende, Vigo (Pontevedra) 36310, Spain

**Keywords:** cancer, immunotherapy, nanotechnology, gene therapy, nanomedicine

## Abstract

Cancer therapy has been characterized throughout history by ups and downs, not only due to the ineffectiveness of treatments and side effects, but also by hope and the reality of complete remission and cure in many cases. Within the therapeutic arsenal, alongside surgery in the case of solid tumors, are the antitumor drugs and radiation that have been the treatment of choice in some instances. In recent years, immunotherapy has become an important therapeutic alternative, and is now the first choice in many cases. Nanotechnology has recently arrived on the scene, offering nanostructures as new therapeutic alternatives for controlled drug delivery, for combining imaging and treatment, applying hyperthermia, and providing directed target therapy, among others. These therapies can be applied either alone or in combination with other components (antibodies, peptides, folic acid, *etc.*). In addition, gene therapy is also offering promising new methods for treatment. Here, we present a review of the evolution of cancer treatments, starting with chemotherapy, surgery, radiation and immunotherapy, and moving on to the most promising cutting-edge therapies (gene therapy and nanomedicine). We offer an historical point of view that covers the arrival of these therapies to clinical practice and the market, and the promises and challenges they present.

## 1. Introduction

Chemotherapy, surgery and radiotherapy are the most common types of cancer treatments available nowadays. The history of chemotherapy began in the early 20th century, but its use in treating cancer began in the 1930s. The term “chemotherapy” was coined by the German scientist Paul Ehrlich, who had a particular interest in alkylating agents and who came up with the term to describe the chemical treatment of disease. During the First and Second World Wars, it was noticed that soldiers exposed to mustard gas experienced decreased levels of leukocytes. This led to the use of nitrogen mustard as the first chemotherapy agent to treat lymphomas, a treatment used by Gilman in 1943. In the following years, alkylating drugs such as cyclophosphamide and chlorambucil were synthesized to fight cancer [1,2]. Kilte and Farber designed folate antagonists such as aminopterin and amethopterin, leading to the development of methotrexate, which in 1948 achieved leukemia remission in children [3]. Elion and Hitchings developed 6-thioquanine and 6-mercaptopurine in 1951 for treating leukemia [4,5]. Heidelberger developed a drug for solid tumors, 5-fluorouracil (5-FU), which is up to now an important chemotherapy agent against colorectal, head and neck cancer [6]. The 1950s saw the design of corticosteroids, along with the establishment of the Cancer Chemotherapy National Service Center in 1955, whose purpose was to test cancer drugs. At that time, monotherapy drugs only achieved brief responses in some types of cancers [7]. By 1958, the first cancer to be cured with chemotherapy, choriocarcinoma, was reported [8]. During the 1960s, the main targets were hematologic cancers. Better treatments were developed, with alkaloids from vinca and ibenzmethyzin (procarbazine) applied to leukemia and Hodgkin's disease [9-11]. In the 1970s, advanced Hodgkin's disease was made curable with chemotherapy using the MOMP protocol [12,13], which combined nitrogen mustard with vincristine, methotrexate and prednisone, and the MOPP protocol [14,15], containing procarbazine but no methotrexate. Patients with diffuse large B-cell lymphoma were treated with the same therapy and, in 1975, a cure for advanced diffuse large B-cell lymphoma was reported using protocol C-MOPP, which substituted cyclophosphamide for nitrogen mustard [16].

Surgery and radiotherapy were the basis for solid tumor treatment into the 1960s. This led to a plateau in curability rates due to uncontrolled micrometastases. There were some promising publications about the use of adjuvant chemotherapy after radiotherapy or surgery in curing patients with advanced cancer. Breast cancer was the first type of disease in which positive results with adjuvant therapy were obtained, and also the first example of multimodality treatment, a strategy currently employed for treatment of numerous types of tumors. In the late 1960s, the use of adjuvant chemotherapy changed the concept of localized treatment.

There was significant progress in 1978 when higher cure rates of metastatic germ cancer were achieved by combining cisplatin, bleomycin and vinblastine [17-19]. The experience with polychemotherapy in hematologic cancer brought to light the fact that different drugs act against tumor cells in different phases of their cellular cycle. One of these solid tumor drugs was CMF (cytoxan, methotrexate and fluorouracil), a standard therapy for treating breast cancer for over 30 years. Understanding of molecular changes in cancer cells quickly developed after the 1970s. As a consequence, numerous drugs with various mechanisms of action were introduced during the 1980s. Subsequent advances and developments led to liposomal therapy, which places drugs inside liposomes (vesicles made of lipid bilayers), decreasing some of the side effects of chemotherapy such as cardiotoxicity. Examples of liposomal drugs include liposomal doxorubicin and daunorubicin, one of the first steps in nanotechnology-based approaches. The 1990s sparked the beginning of targeted chemotherapy by screening for specific critical molecular targets. These advances in modern chemotherapy and studies on genetics and molecular biology contributed to the ongoing decline in death rates. Data from the genome sequence suggested that many dysfunctions associated with cancer could be due to the abnormal function of some protein kinases. The current pharmacological trend has been to develop kinase inhibitors [20,21]. The first tumors targeted with drugs approved by the FDA (Food and Drug Administration) and the EMEA (European Medicines Agency) were renal cell cancer, hepatocellular cancer and gastrointestinal stromal tumors. In recent years, numerous specific tumors have been tested with various kinase inhibitors and there is a trend towards combining chemotherapy with these new targeted therapies.

Chemotherapy is curative in some types of advanced cancer, including acute lymphoblastic and acute myelogenous leukemia, Hodgkin's and non–Hodgkin's lymphoma, germ cell cancer, small cell lung cancer, ovarian cancer and choriocarcinoma. In pediatric patients, curable cancers include acute leukemia, Burkitt's lymphoma, Wilms' tumor and embryonal rhabdomyosarcoma. Although treatment is not always curative for these cancers, there has been significant improvement in progression-free and overall survival. Another modality of treatment is neoadjuvant therapy, which aims to reduce the size of the primary tumor and prevent micrometastases. This type of treatment improves on more conservative surgical techniques in preserving the functionality of important organs. Neoadjuvant chemotherapy is indicated for anal, breast, lung, gastroesophageal, rectal, bladder and head and neck cancer, as well as some types of sarcoma. There are many cancers for which adjuvant chemotherapy has been established with curative effect, and with the new effective drugs and combinations the curability rates are expected to rise even more. Since 1990, the incidence and mortality of cancer have been declining and despite the increase in the elderly population [22], mortality rates for the United States declined from 2005 to 2007.

In 1890, Halsted performed the first radical mastectomy, believing that cancer would be more curable if surgical techniques were more aggressive, thus avoiding regional recurrences. He had many followers at that time, but thanks to advances in chemotherapy, radiotherapy, biology and technology, the outlook now is quite different. Radical surgery has now been replaced by less extensive operations.

The turn of the 20th century marked the beginning of the development of cancer surgery techniques, with the first abdominoperineal resection performed in 1908 by Miles [23], the first lobectomy being performed in 1912 [24,25] and the first radical hysterectomy performed by Wertheim in 1906, all carried out under oncological criteria. Additionally, in 1904, Young made the first radical suprapubic prostatectomy. Modern surgery has changed significantly, with Halstedian techniques replaced by non-invasive procedures such as laparoscopic colectomy (for the removal of colon cancer) [26], videothoracoscopy, radiofrequency ablation and radiosurgery techniques such as Cyberknife® [27]. Breast-conserving surgery with sentinel-node removal has been used to improve esthetic results and avoid lymphedema [28]. Another example of conservative surgery is the use of laryngoscopic laser surgery in early laryngeal cancer [29]. The most recent development is the Da Vinci®, a robotic system for the removal of cancer from prostate and kidney [30].

The discovery of X-rays and radiation by Becquerel and Rontgen in the late 19th century was the first step towards radiation treatment. Marie Curie's work greatly contributed to the development of radiotherapy. The first cancer case cured exclusively by radiation occurred in 1898. After World War II, technological progress allowed charged particles to be propelled through a vacuum tunnel called linac, or linear accelerator. In 1960, Ginzton and Kaplan began to use a rotational linac radiotherapy called “Clinac 6”, which was used to concentrate X-rays more deeply thereby they not affecting the skin as much. The development of modern computers enabled three-dimensional X-ray therapy, such as intensity-modulated radiation therapy (IMRT) using mapping information from Computed Tomography (CT) scans. This provides a three-dimensional reconstruction, which helps avoid toxicity since the contours of the tumor are targeted and separated from healthy tissues. In 2003, a specific type of IMRT was developed called the TomoTherapy® system. This treatment uses CT-guided IMRT technology that directs the radiation source by rotating it around the patient, which makes the morphological limits of a tumor easier to trace with the beam [31]. Another significant trend is the use of charged particle radiotherapy with proton or helium ions for specific types of patients with melanoma of the uveal tract. It is also used as adjuvant therapy for skull base chondroma, chondrosarcoma and spine (usually cervical). In summary, the lines of development have been fractionated dose delivery, technological advances in X-ray production and delivery and improvement of computer-based treatment planning.

The latest advance in scanning technology with radiotherapy therapy is four-dimensional (4D) conformal radiotherapy [32], which records a video sequence of tumor movement. This therapy uses dynamic CT images of the body that compensate for any movement by the target, including movements when patients breathe. There are two forms of this therapy: Image-guided radiation therapy (IGRT) and Image-guided adaptive radiation therapy (IGART).

Another combined system is radiogenic therapy, which induces the formation of cytotoxic agents against cancer cells. Lower doses of radiation are used along with a biological agent, and stimulation by radiation produces cytotoxic agents. This complex technology was developed to use radiation to activate promoters and thus inducing the expression of genes responsible for producing enzymes. These proteins activate the selected drug, and the activated form of the drug then destroys cancer cells. Another modality consists of radiolabeled molecules, which fight cancer by delivering targeted radiation to specific receptor-bearing cells. Radioactive isotopes (Iodine-125 or Indium-111) emit Auger electrons, which have the potential to be delivered to specific sets of target cells, thus sparing healthy cells.

This manuscript reviews the evolution of oncological treatments available today, together with several immunotherapeutic approaches and nanoscale-based therapeutics including successes, drawbacks and recent progress.

## 2. Immunotherapy

The concept of Immunotherapy in medicine incorporates the use of components of the immune system, including antibodies (Abs), cytokines, and dendritic cells, to treat various illnesses, such as cancer, allergies, and autoimmune and infectious diseases. Immunotherapy also includes the use of vaccines for the prevention of allergies and tumors. Immunotherapy adds new dimensions to clinical practice, offering much more specificity, higher efficacy, directed therapy, less toxicity, lower secondary effects and better tolerance.

Although immunotherapy can be used for several illnesses (macular degeneration, autoimmune diseases, *etc.*), in the case of cancer, the aim of immunotherapy is to kill tumor cells (either directly or indirectly) or to help patients' immune systems destroy tumors. Of all the types of anti-tumoral immunotherapy, this review will focus on the use of antibodies, their history, problems and current applications.

### 2.1. Antibodies: History

Antibodies (Abs) are one of the most important defense mechanisms for vertebrate animals. They are produced by B cells, which, after antigen-mediated activation, undergo differentiation to secretory (plasma) cells thus producing soluble antibodies. Antibodies are highly specific, and they recognize and eliminate pathogens and disease antigens, but can be deliberately generated to recognize different target molecules (tumor markers, bacteria, receptors, cytokines, hormones, *etc.*). Thus, Abs can be used in many applications, including diagnostic techniques, research and therapy (against infections, tumors, transplants and autoimmune diseases).

**Figure 1.**
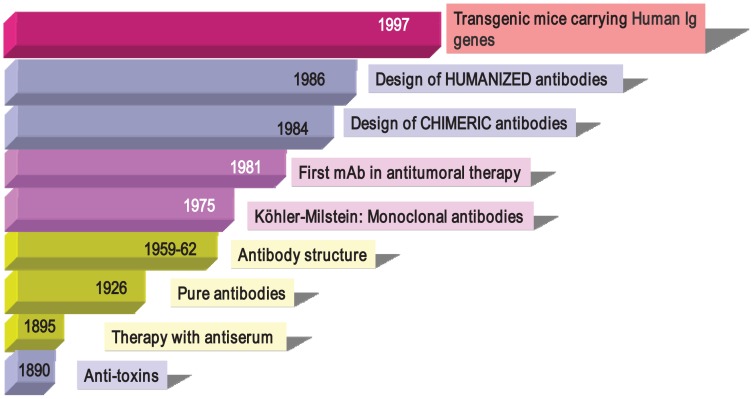
History of antibodies. In 1890 von Behring and Kitasato showed that it was possible to generate anti-toxins (against tetanous, diphtheria), and soon after, therapy with antiserum containing antitoxins were used in patients. It took several years to purify the antibodies (1926) and even more to know their structure. On 1975, Milstein and Köhler developed the first monoclonal antibody, and the generation and application of monoclonal antibodies started (on diagnosis, research and therapy), initiating the Modern Immunology. In the 1980s, the first anti-tumoral monoclonal antibody was tested and molecular biology techniques started to designed chimeric and humanized antibodies. Later on, transgenic mice carrying human Ig genes and other animal models were used to produce fully human antibodies.

Antibodies were described in 1890 (Figure 1) by von Behring and Kitasato as “anti-toxins” that appeared in the serum of animals after immunization with inactivated toxins (toxoids) [33]. The researchers noted that protection could be transferred to other animals through the use of these antisera, thus beginning what it is known as “serum therapy” for treating infectious diseases (diphtheria and tetanus) in humans. Soon after, these sera elements were described as “anti-bodies” because they could be directed not only against toxins, but also against a large variety of organisms and compounds (bacteria, proteins, chemicals, *etc.*). Immunotherapy initially began with the use of antisera obtained from animals such as horses and sheep containing, among other things, a mixture of antibodies from the activation of different B cell clones, so-called “polyclonal antibodies” (PAbs). In 1926, Felton and Bailey obtained pure antibodies, but it was not until the 1960s, thanks to the work of Porter and Edelman (1972 Nobel Prize winners), that the Ab structure became known. After the introduction of Abs to therapy, researchers observed that the transferred defense was only temporary (as opposed to vaccination, which induces long-term memory). In addition, it often incurred anaphylactic responses that were occasionally fatal and which greatly reduced their use in human therapy. However, these problems did not prevent PAbs from being used successfully in diagnostictechniques and even in preventive therapies. Anti-snake venom, ant-tetanus and anti-Rh+ gamma globulins are still being used in clinical practice.

In 1975, Cesar Milstein and George Köhler (1984 Nobel Prize winners) succeeded in generating monoclonal antibodies (mAbs) by fusing mouse B cells with B cell tumors (myeloma) to create hybrid cells, which were immortal and had the capacity to produce large quantities of a single (monoclonal) antibody [34]. In 1976, genetic studies by Susumu Tonegawa revealed the basis for the vast diversity of antibodies, identifying the process of somatic recombination in immunoglobulin genes [35]. Since the publication of the monoclonal antibody technique, mouse and rat mAbs have been used in many laboratories with thousands of applications in various scientific fields, in diagnostic techniques (clinical, food, environmental), research and in therapy (antitumor, autoimmune diseases). Monoclonal antibodies have helped in the discovery of new molecules (such as the identification of more than 300 membrane proteins, grouped under the CD concept or Cluster of Differentiation), transcription factors, viral, plant and bacterial proteins, phosphorylated compounds involved in death by apoptosis, factors involved in enzymatic cascades and many more. As an example of their usefulness, the current classification of leukemia by the World Health Organization is based on the presence or absence of membrane molecules recognized by monoclonal antibodies that define leucocyte populations in various stages of differentiation.

But one of the greatest achievements with monoclonal antibodies is their use in human therapy. Surgery, chemotherapy and radiotherapy are not specifically directed to tumor cells and may also affect healthy tissue. Antibodies can provide specificity and lower toxicity, opening new therapeutic possibilities. The first evidence of this potential came in 1982 when a patient suffering from lymphoma responded to treatment using a mouse mAb directed specifically against his tumor B lymphocytes [36]. This response rapidly encouraged research into the production of potentially therapeutic Abs. However, clinical trials results revealed that many patients receiving this therapy developed an immune response directed against the therapeutic Abs, a response known as HAMA (Human Anti-Mouse Antibodies) or HARA (Human Anti-Rat Antibodies). Some even developed anaphylactic reactions, especially after repeated administration. The high immunogenicity of antibodies due to their large size compared to conventional pharmaceutical drugs, and differences in the pattern of glycosylation between murine and human Abs, once again led to the cessation of antibody use in therapy.

**Figure 2.**
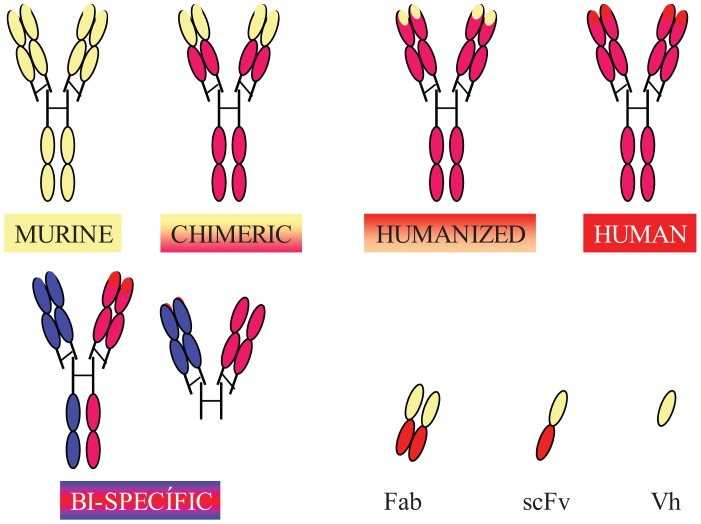
Several antibody molecules and some antibody fragments are shown. Chimeric (mouse-human) antibodies carry mouse heavy and light variable domains (in yellow) being the rest of the molecule of human origin (in red). In the case of humanized antibodies, only the hypervariable regions are mouse derived (in yellow). It is possible to generate bi-specific antibody molecules, using different heavy and light chains (each arm will have a different specificity). Fab: fragment antigen binding; scFv: single chain Fragment variable; Vh: variable domain from the heavy chain.

Completely human mAbs needed to be developed to avoid immune rejection, but their production was much more complex than initially thought. In contrast to mouse or rat myeloma cells, human myeloma cells proved difficult to adapt to continuous growth *in vitro*. Researchers tried to resolve this problem by immortalizing B cells using the Epstein-Barr virus (EBV) [37] and by fusing human B cells with well-established murine myeloma (obtaining heterohybridomas) [38]. However, the low production of antibodies in these cells, the instability of heteromyeloma cells and numerous technical problems lead to the search for alternative methods for generating human-like mAbs in the mid-1980s. One of these methods was the modification of murine mAbs through genetic engineering (Figure 2).

Chimerization (murine variable domains linked to constant regions of human heavy and light chains), humanization (only hypervariable regions of murine origin), primatization (chimeric structure of human and primate origin) and the design of recombinant antibody fragments, such as Fv (variable fragment), Fab (antigen binding fragment), scFv (single chain variable fragment) and minibodies (artificial polypeptides with a structure based on the IgV domain), are some of the methods that have been used over the last 30 years to reduce antigenicity and maintain the binding affinity and specificity of the original Ab. Rituximab, a chimeric anti-CD20 mAb, was the first mAb approved by the FDA for antitumor therapy. However, a year earlier, several mAbs conjugated with radioactive elements were approved for *in vivo* tumor detection. Every year since then, several mAbs have been approved for therapy in the US and Europe, and more than half of them are chimeric or humanized mAbs (See Table 1).

In addition to fully engineered antibodies, antibody fragments also have advantages compared to whole antibodies, especially in terms of the rate of solid tumor penetration. Jainr [39] determined that an intact IgG molecule needed 54 hours to move 1 mm into a solid tumor, whereas a Fab fragment reached the same distance in 16 hours. While the expression of chimeric and humanized antibodies was carried out in eukaryotic hosts, such as mammalian or plant cells, bacteria have been the most widely used organism for the production of recombinant antibody fragments [40-42]. However, despite numerous advantages, such as avoiding animal immunization and hybridoma production, their low cost and easier production [43], antibody fragments have shorter circulating half-lives compared to full-size antibodies, lack glycosylation and lack effector functions due to the absence of their Fc region (unless added). Thus, antibody therapies using incomplete antibodies have been relegated to those cases where rapid elimination of antibodies from the blood is required and to local therapy (e.g., macular degeneration). Modified versions, such as PEGylation of fragments (modification of a molecule by linking of one or more polyethylene glycol chains) [44] to improve circulation half-life, glycosylation and Fc region engineering are some of the recent approaches used by researchers to overcome these problems [45].

In the mid 1990s, thanks to the development of molecular biology techniques and microinjection and manipulation of embryonic cells, several groups created various transgenic mice models carryinghuman Igs genes (Figure 1). The introduction of human Ig loci in these mice was carried out using various vectors, such as miniloci, yeast and human artificial chromosomes (YACs and HACS, respectively) and P1 vectors. Transgenic mice can be immunized with almost any Ag (including human tumor cells), and their spleens can be used to obtain hybridomas following the conventional protocol [46-49]. Moreover, mice can produce human Abs of intermediate/high affinity because they can introduce mutations in their human Igs transgenes through the mechanism of somatic hypermutation. Fully human monoclonal antibodies show several advantages in human therapy, which include low or no immunogenicity, better interaction with human effector systems and patterns of glycosylation and a longer half-life in human serum. In recent years, many fully human mAbs have been introduced into clinical trials and some of them have been approved by regulatory agencies (Table 1).

In addition to the use of transgenic mice to generate fully human mAbs, other alternatives have been developed, such as the use of immunodeficient mice receiving human hematopoietic tissue, the use of chicken eggs with human Ig coding genes inserted into embryonic cells and the generation of transgenic tobacco plants for producing human mAbs.

Moreover, several groups are working on modifications of the basic antibody structure to generate monovalent and multispecific reagents that may have various therapeutic properties and even completely new structures. Examples of these new reagents include antibody alternative protein scaffolds based on leucine-rich repeat molecules of lamprey variable lymphocyte receptors (VLRs), libraries of fibronectin domains and designed ankryin repeat proteins (DARPs) [50]. With all these novel antibody formats, immunogenicity, stability and aggregation problems should be carefully considered.

### 2.2. Timing: From Development to Clinic

Soon after mAbs generation was reported in 1975, the potential of mAbs became clear and many companies showed interested in developing new reagents for diagnosis and designing new equipment, among other contributions. However, when it came to the field of human therapy, pharmaceutical companies did not initially show much interest in the development of monoclonal antibodies, although several research groups were showing promising results in preclinical and clinical studies. The reasons for their reluctance are many:

1. A number of pharmaceutical companies had experience with generating small compounds, most of them chemically synthesized, but not with generating large biological molecules produced by cells. Moreover, sophisticated equipment and cell culturing under controlled conditions, with full quality assurance, are necessary for antibody production.
2. There was the perception by pharmaceutical companies that production of mAbs was not going to yield sufficient profit. Most companies preferred to concentrate their efforts on developing analogues of well-known drugs rather than on new products, while at the same time most clinicians opted for trials using combinations of known agents. This view took years to change. Advances in mAb engineering helped develop more effective mAb drugs with high specificity, improved potency and stability and decreased immunogenicity, which helped change the companies' initial reluctance.
3. In terms of clinical trials, there were concerns about the cost of the trials (around 10 times more expensive today than 30 years ago), the time required for preclinical pharmacology and toxicology studies (which are much more regulated) and the difficulty in conducting early clinical trials. Since new drugs can only be tested against advanced and usually heavily pretreated disease, it is unlikely that dramatic responses will occur with these patients.
4. The requirement for fetal calf serum in cell hybridoma cultures introduced another problem when Mad Cow Disease was identified in the early 1990s. The FDA proposed a limit on materials used in some medical products in order to keep them free of the agent thought to responsible for Mad Cow Disease (also known as bovine spongiform encephalopathy or BSE), making it necessary to find alternatives, such as enriched media without serum.

### 2.3. Current Antibodies Used in Cancer Therapy

Since 1988, 228 mAbs have entered clinical studies for various diseases, with 56% of those currently in clinical development. Some of these mAbs are listed in Table 1. The first mAb approved for cancer therapy was rituximab (Rituxan™), a chimeric antibody directed against CD20, for non-Hodgkin's lymphomas. Since then, many others have reached the market, including those for the treatment of breast cancer (trastuzumab, Herceptin®), acute myeloid leukemia (gemtuzumab Ozogamicin, Mylotarg™), chronic lymphocytic leukemia (alemtuzumab, Campath-1H®), colorectal tumor (cetuximab, Erbitux™) and several types of cancer (bevacizumab, Avastin™). Companies such as Genentech Inc., Amgen, Bristol-Myers-Squibb, Imclone Systems and Trion Pharma represent only a portion of the pharmaceutical companies involved in the antibody market related to cancer therapy (Table 1).

New developments have also occurred in the immunoconjugate field and many of them are currently being explored by the pharmaceutical industry. Immunoconjugates include antibodies linked to cancer-killing agents such as drugs, cytokines, toxins and radioisotopes. The objective is for the antibody to act as a transporter for the cancer-killing agent, concentrating the agent directly in the cancer cell, with minimal damage to healthy cells. Although conjugated antibodies showed toxicity in the past, more recent approaches under development appear to decrease unwanted side effects. Pharmaceutical companies are developing immunoconjugates independently, forming partnerships with specialized players and even acquiring small biotech companies that are focused on the field of immunoconjugates.

Although the challenge of their potential immunogenicity requires special attention, there are several practical advantages to immunoconjugates over single antibodies. These include lower dosages, which may lead to lower treatment costs and fewer side effects; the reintroduction of antibodies that historically have shown low efficacy in isolation; the possibility of using bacteria or plant cells to produce immunoconjugates rather than using mammalian cell cultures (decreasing costs and complexity) and the large number of potential combinations (antibodies-cancer killing agents) that are possible. The advantages of immunoconjugates over single antibodies make them crucial players in new cancer therapy developments.

### 2.4. Costs Involved in Monoclonal Antibodies and Cancer

Although many researchers have worked on monoclonal antibodies and cancer (close to 60,000 reports on this subject can be found in PubMed (http://www.ncbi.nlm.nih.gov/pubmed) the therapeutic mAb market moved much more slowly than initially expected, due mostly to the problems indicated above. This situation has changed in recent years and mAbs are now the largest class of biological therapies under development, representing a multi-billion dollar worldwide market. As reported recently by Scolnik [51], the 22 mAbs currently marketed in the US have a sales growth rate of 35% compared to less than 8% for small-molecule drugs. Oncology and autoimmune diseases are the most successful indications for these drugs, with five mAbs having sales in excess of $3B. Thanks to basic research, researchers are identifying new biomarkers, which could be potential targets for mAbs. There are currently numerous mAbs at various developmental stages and it is expected that many of them will be available for clinical use in the near future.

## 3. Nanoscale and Nanostructure-Based therapeutics

Chemotherapy, radiation therapy and surgery are the most common types of cancer treatments available today. More recent treatments, which are at various stages of development, include angiogenesis inhibitor therapy, biological therapies (including interferons, interleukins, colony-stimulating factors, monoclonal antibodies, vaccines, gene therapy and nonspecific immunomodulating agents), bone marrow and peripheral blood stem cell transplantation, laser therapy, hyperthermia, photodynamic therapy and targeted cancer therapies [52].

In the last two decades, a large number of nanoscale and nanostructure-based therapeutic and diagnostic agents have been developed, not only for cancer treatment but also for its prevention and diagnosis [53]. Targeted cancer, hyperthermia, photodynamic and gene therapies are just some of the cancer treatments that use engineered nanomaterials. These therapies can be used in isolation or in combination with other cancer treatments, thereby taking advantage of their ability to target tumors (actively or passively), to respond to physical or chemical stimulation (internal or external) and to deliver therapeutic genes to the cell nuclei.

**Figure 3.**
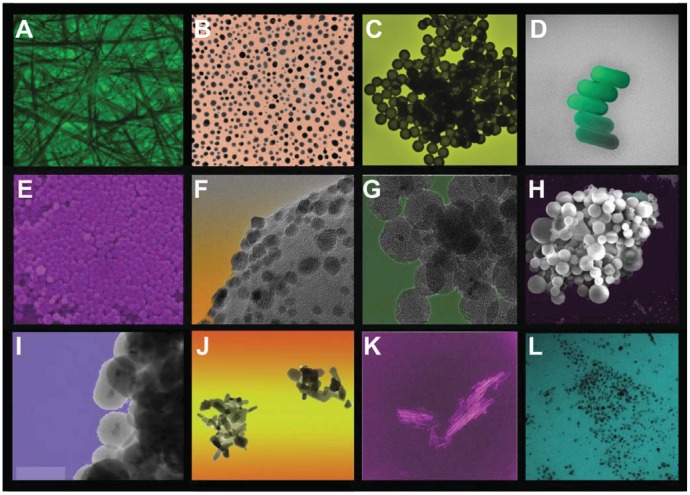
A collection of scanning and transmission electron microscope images (color added) of different nanoscale or nanostructured materials used in biomedicine. (**A**) Silver nanowires; (**B**) gold nanoparticles; (**C**) SiO_2_/Au core/shell nanoparticles (nanoshells); (**D**) gold nanorods; (**E**) dense silica nanoparticles; (**F**) gold nanoparticles on an inorganic support; (**G**) mesoporous silica; (**H**) Poly(lactic-co-glycolic acid) (PLGA) microparticles; (**I**) Fe_3_O_4_/SiO_2_ core/shell nanoparticles; (**J**) ZnO nanoparticles; (**K**) TiO_2_ nanotubes; (**L**) Fe_3_O_4_ nanoparticles.

The main objective of nanomaterials in cancer treatment is to deliver a therapeutic moiety to tumor cells in a controlled manner (depending on the required pharmacokinetic) while minimizing side effects and preventing drug resistance. Nanoscale and nanostructured materials may also be used in diagnosis to detect and prevent pathologies as soon as possible, ideally being able to sense cancer cells and associated biomarkers. Compared to conventional therapies, nanoparticles show six clear advantages in cancer treatment and/or diagnosis: (1) they can be synthesized in specific sizes and with surface characteristics to penetrate tumors by taking advantage of the enhanced permeation and retention effect (EPR) (a mechanism known as passive targeting); (2) they can be engineered to target tumor cells by surface functionalization with biomolecules that attach to tumor-specific cell markers (a mechanism known as active targeting); (3) they can be engineered to penetrate cells and physiological barriers (e.g., blood-brain barrier, blood-retinal barrier); (4) they can increase the plasma half-life of carried chemotherapeutic drugs, which are usually highly hydrophobic; (5) they can protect a therapeutic payload from biological degradation; and (6) they can be synthesized as multifunctional platforms for combined imaging and therapeutic applications (theragnostic nanoparticles). Examples of various nanostructured materials with potential applications in oncology are shown in Figure 3.

**Figure 4.**
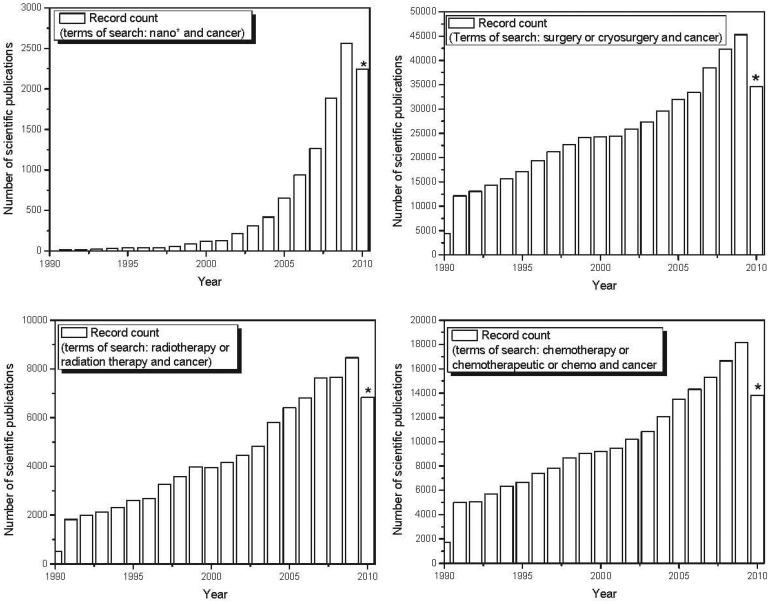
Temporal evolution in the number of scientific papers published involving nano-based applications developed to fight cancer in the last decade. Document types include articles, reviews, meeting abstracts, patents, editorials, letters and news. (Source: ISI Web of Knowledge © The Thomson Corporation. Date of search: December, 2010.)*2010 indexing was incomplete at the time of search.

The advantages of biocompatible nanomaterials have contributed to their significant expansion in cancer treatment. Targeted therapies for oncology are predicted to reach a 30 billion euro global market by 2015 [54]. The total market for nanobiotechnology products reached as high as $19.3 billion in 2010 [55]. Table 2 compiles some of the clinically approved nano-based therapeutics for cancer treatment and diagnosis. Many other nanoscale or nanostructure-based therapeutic and diagnostic agents are currently in clinical trials at various stages of development. In 2008, Zhang *et al.* [53] reported on 15 clinical trials being conducted for nanoparticle-based therapeutics. A year later, 50 ongoing clinical trials using nanoparticles for cancer were mentioned by Bergin [55], and at present, there are more than 70 clinical trials under development [56]. This large number of commercial

nano-based therapeutics for use in cancer treatment is also reflected in the exponential increase in scientific publications and patents involving nanomaterials in recent years. Figure 4 shows the evolution over the last decade in the number of published scientific papers and issued patents involving nano-based applications developed to fight cancer. The number of papers and patents involving traditional forms of therapy (chemotherapy, radiation therapy and surgery) grew linearly over the last decade. However, the use of the terms “nano-” and “cancer” has shown exponential growth over the past decade, demonstrating a major focus on nano-based tools applied to cancer treatment and diagnosis. Recent advances in the use of nanoscale and nanostructured-based therapeutic agents in cancer treatment are reported below.

### 3.1. Targeted Cancer Therapies

Nanoparticles are engineered to achieve cell targeting by using selective moieties (e.g., antibodies and their fragments, carbohydrates, peptides, nucleic acids), which binds to its corresponding antigen, cell surface carbohydrate or over-expressed receptor in tumor cells. The rapid cellular proliferation of these cells is also exploited by coupling the nanoparticles with different biological agents, such as folic acid. The rationale for coupling these carriers with folic acid is that the folate receptor is over-expressed in a broad range of tumor cell types, including solid and hematological malignancies [57]. Once it has reached the target, the cargo is released into the interior of the cell, and ideally, a signaling marker attached to the vector will aid the physician in visualizing the tumor. Such a vector may also be grafted with a moiety (usually PEG), which retards recognition by the reticulo-endothelial system (RES) to increase nanoparticle systemic circulation. In addition to recognition moieties, carried drugs and signaling elements attached to nanoparticles, numerous authors have also envisioned and designed vectors with additional functionalities, including cell-penetrating moieties, combinations of several drugs, combinations of drugs and genes, prodrugs (which become drugs upon biochemical modification by tumor cells), stimulus-sensitive agents that can be externally triggered and molecules for evaluating therapeutic efficacy. The more functionality added to the vector, the better the chances of reaching the target; however, its chances of being detected by the RES also increase. Therefore, currently marketed nanoparticles use passive targeting and active targeted nanoparticles are still being developed. Examples of active targeted nanoparticles are reviewed elsewhere [58].

Targeted nanoparticle fabrication remains a challenge due to the multiple steps involved, which include biomaterial synthesis and assembly, targeting ligand coupling/insertion, drug loading, surface stabilization and final purification, which could cause batch-to-batch variations and, therefore, quality concerns. For this reason, single-step synthesis of targeted nanoparticles by self-assembling pre-functionalized biomaterials provides a simple and scalable manufacturing strategy [59]. Mass production is also a serious concern and continuous synthesis procedures are therefore still being sought. When using batch reactors to synthesize nanoparticles, several drawbacks usually appear, including: (1) heterogeneous distribution of reactants and temperatures in the reactor; (2) insufficient mixing; (3) variations in the physicochemical characteristics of products from different batches; (4) their inherent discontinuity; and (5) the numerous post-synthesis purification steps that are usually required. In order to overcome these disadvantages, microfluidic reactors (e.g., micromechanized micromixers, capillaries, junctions) have been used in the continuous synthesis of nanoparticles to precisely control reaction temperatures and residence times, thereby rendering nanoparticles with narrow particle-size distributions. Other continuous synthesis processes are usually preferred when synthesizing nanoparticles on a large scale (e.g., laser pyrolysis, arc discharge methods).

Another concern is the adaptive response of the immune system after repeated applications of nanoparticles. Immunological memory, created from the primary response to a specific nanoparticle, provides an enhanced response to secondary encounters with the same type of nanoparticle. As an example, the recognition of PEGylated liposomes by anti-PEG antibodies has been reported to occur between 2 to 4 days after the first administration of PEG-liposomes, leading to fast clearance from circulation [54]. Finally, one of the last major barriers to achieving the transition of targeted nanoparticle use into clinical practice is the complete understanding of potential toxicological properties of these materials, along with their exact pharmacodynamics and pharmacokinetics.

In spite of these hurdles, many research groups are focusing their efforts on solving them. Other groups are also directing their efforts towards designing more efficient targeted nanoparticles for cancer treatment in terms of structure, morphology, biocompatibility and surface functionalization. Some of those advances will be described later in this document.

Novel targeted theragnostic nanoparticles have been synthesized and their bi-functionality demonstrated. Among them are perfluorocarbon nanoemulsions, which are in clinical trials [60]. Quain *et al.* [61] coupled PEGylated gold nanoparticles to a single-chain variable fragment antibody, which recognized the epidermal growth factor receptor overexpressed in many types of malignant human tumors, and demonstrated the targeting capabilities of these vectors in nude mice bearing human head-and-neck tumors. The nanoparticles were also able to function as tags for spectroscopic detection with surface-enhanced Raman spectroscopy. Magnetic targeting has also been used as a physical method for targeting and visualizing tumors. Effects of magnetic targeting on the extent and selectivity of nanoparticle accumulation in tumors of rats harboring orthotopic 9L-gliosarcomas were analyzed using magnetic resonance imaging (MRI) [62]. Sun *et al.* also demonstrated the targeted drug release capabilities of iron oxide nanoparticles conjugated with a drug (methotrexate) and a targeting ligand, chlorotoxin, while monitoring tumor-cell specificity *in vivo* using MRI [63]. Weng *et al.* demonstrated the targeted tumor cell internalization and imaging of multifunctional quantum dot-conjugated immunoliposomes, *in vitro* and *in vivo* [64]. In this targeted delivery system, anti-HER2 single chain Fv fragments were attached to the end of PEG chains located on the surface of liposomes.

Targeting via extracellular activation of the nanocarrier is a promising method for achieving active targeting using physiological stimuli present in the tumor environment. Triggering mechanisms that only release the transported cargo of nanocarriers into the tumor environment take advantage of its acidic pH and uncontrolled enzyme production. A complete description of these systems is reported elsewhere [58].

Tumor targeting of prodrugs that become active once they reach tumor cells is another novel strategy for avoiding unwanted side effects of the drug, and it allows for the delivery of large doses of drugs. Following this approach, Dhar *et al.* [65] synthesized Pt(IV)-encapsulated prostate-specific membrane antigen targeted nanoparticles of poly(D,L-lactic-co-glycolic acid) (PLGA)-poly(ethylene glycol) (PEG)-functionalized controlled release polymers. After reduction in the interior of the tumor cells, the prodrug becomes cisplatin, which cross-links on nuclear DNA.

### 3.2. Photodynamic Therapy

Photodynamic therapy (PDT) is a technology that uses a photosensitizer that is activated upon exposure to visible or near infrared (NIR) light, and transfers energy to molecular oxygen, thereby generating reactive oxygen species (e.g., singlet oxygen, free radicals, peroxides). The subsequent oxidation of lipids, amino acids and proteins induces cell death. A complete review of photosensitizers is reported elsewhere [66]. FDA-approved photosensitizers absorb in the visible spectral regions below 700 nm, where light penetrates only a few millimeters into the skin. PDT istherefore limited to treatment of certain types of skin cancer and its effectiveness for other tumors is not yet apparent [66,67]. PDT is usually performed as an outpatient procedure and may be repeated and used in combination with other therapies, such as surgery, radiation and chemotherapy [52].

Photosensitizers are susceptible to photobleaching under light irradiation, and have therefore been loaded within nanoparticles to avoid this drawback. Most photosensitizers are also highly hydrophobic, so nanoparticles are being explored as carriers to increase their bioavailability. Noble metal nanoparticles have proven very useful as agents in photodynamic therapy due to their enhanced absorption cross sections, which are four to five orders of magnitude larger than those offered by conventional photoabsorbing dyes [68]. Silica nanoparticles synthesized in the non-polar core of micelles have been used to entrap the water-insoluble photosensitizing anticancer drug 2-devinyl-2-(1-hexyloxyethyl) pyropheophorbide. Upon NIR light irradiation, nanoparticles embedded in HeLa cells generate singlet oxygen, resulting in a reduction in the percentage of cell survival [69]. Many other photosensitizers have been embedded within inorganic nanoparticles for PDT, including *meta*-tetra(hydroxyphenyl)-chlorin (*m*-THPC) [70]. A complete review of various nanoparticulate-based carriers for PDT is reported elsewhere [71]. Preclinical studies will determine the added translational value of PDT therapies using photosensitizers loaded into these novel nanoparticles prior to their use in clinical settings.

### 3.3. Hyperthermia

Hyperthermia, as an anticancer therapy, consists of heating a tumor to inhibit proliferation of cancer cells with the aim of destroying or rendering them more sensitive to the effects of conventional protocols of radiation and chemotherapy. In fact, hyperthermia is currently used as an adjunct therapy to radiotherapy and/or chemotherapy. When cells are heated beyond their normal temperature they can become sensitized to conventional therapeutic agents such as radiation and chemotherapy. When high temperatures are used, typically above 43 °C, the heat causes irreparable damage and results in tumor cell death in a process known as thermal ablation. The success of local thermal ablation consists of destroying the entire tumor mass without damaging adjacent vital structures. This requirement is particularly important for patients with limited reserves of tissue function.

Hyperthermia treatments make use of microwaves, ultrasounds and radiofrequency, which can be focused and used locally to target the tumor. A significant advantage of thermal technology is that it is minimally invasive. Mild heat increases blood flow in the tumor, allowing chemotherapy to exert greater effect on cancer cells. By depressing the metabolic activity of target cells, heat also reduces the oxygen demand in the tumor and tumor tissue oxygenation increases, which makes hyperthermia one of the most potent radiosensitizers available [72]. Results from clinical trials conducted under quality assurance guidelines have shown hyperthermia to be beneficial in the treatment of several types of solid tumors, including breast cancer, melanoma, sarcoma and locally advanced cervical cancer, with reports demonstrating improved overall survival, as compared to patients who only receive radiotherapy or chemotherapy [73-75]. It is widely accepted that the benefits of hyperthermia will significantly increase with refinements in heating delivery technologies as well as in monitoring strategies that ensure optimal thermal dose coverage, resulting in advanced local tumor control and prolongation of overall survival. Integration of hyperthermia with emerging imaging technologies, such as non-invasive MR-based thermometry, will help unveil the full potential of hyperthermia for treating cancer.

Nanotechnology may offer a window of opportunity to improve heat delivery. For example, highly focused ultrasound energy transfer to deep brain tumors may be difficult to achieve due to the skull's electromagnetic barrier. Magnetic Fluid Hyperthermia (MFH) uses iron oxides as a heating source due to their excellent magnetic properties and good compatibility [76]. Depending on the route of administration, magnetically mediated hyperthermia can be classified into two main types: arterial embolization hyperthermia, where arterial supply is used to deliver magnetic particles into the tumor tissue, and direct intratumoral injection hyperthermia. Magnetic nanoparticles for hyperthermia settings show the advantage of being able to achieve site-specific tumor targeting through the aid of an external magnetic field. Magnetic nanoparticles can also be simultaneously traced using MRI. These nanoparticles are then selectively heated by application of a high frequency alternating magnetic field. Magnetic energy dissipation from the nanoparticles (Brown and Néel relaxations) induces heating, which produces cell death at temperatures above 43 **°**C. Significant antineoplastic effects of MFH treatment were initially observed in animal models of glioma [77] and prostate cancer [78]. Consequently, Phase I and II clinical trials with thermotherapy using magnetic particles have been conducted to treat prostate carcinoma [79] and glioblastoma multiforme [80,81]. It has been demonstrated that magnetic hyperthermia in conjunction with a reduced radiation dose leads to longer survival following diagnosis of first tumor recurrence compared to conventional therapies in the treatment of recurrent glioblastoma [81]. Limiting factors of magnetic hyperthermia have been reported, including patient discomfort at high magnetic field strengths as well as irregular intratumoral heat distribution even upon direct intratumoral injection [82].

Magnetoliposomes, *i.e.*, magnetic nanoparticles encapsulated within liposomes, have been designed to achieve active targeting of tumor cells by electrostatic interaction before hyperthermia treatment [83]. Other active strategies, including antibody-functionalized magnetoliposomes, have been used in combination with hyperthermia, demonstrating effective targeting and cytotoxic responses when applying alternating magnetic fields in tumor-bearing mouse models [84].

The harnessing of therapeutic effects of nanoparticle-driven hyperthermia will likely take advantage of the feasibility of using these vectors to load drugs or biological agents and trigger their release upon heating, in order to increase tumor control and disease-free survival. The use of magnetic hyperthermia to trigger drug release has also been demonstrated as feasible in combinatorial approaches for cancer treatment. Purushotham *et al.* [85] developed magnetic nanoparticles coated with a thermoresponsive polymer poly-*n*-isopropylacrylamide (PNIPAM). With these nanoparticles, simultaneous hyperthermia and drug release of therapeutically relevant quantities of doxorubicin at hyperthermia temperatures was achieved *in vitro. In vivo* targeting of those doxorubicin-loaded nanoparticles injected directly via the main hepatic artery to hepatocellular carcinoma in a rat model was followed by MRI examination.

NIR-absorbing nanoparticles have the advantage of being able to absorb or scatter light, thus producing heat, which increases the temperature in the tissue where the nanoparticles have been embedded. This region of the electromagnetic spectrum is notable for minimal absorption by water and biological chromophores [86]. Therefore, NIR light is preferable as a trigger in biomedical applications because it has maximal penetration of tissues due to their minimal absorbance at those wavelengths [87]. Hemoglobin and water, the major absorbers of visible and infrared light, respectively, have their lowest absorption coefficient in the NIR region (around 650–900 nm). NIR light has been shown to travel at least 10 cm through breast tissue and 4 cm through skull/brain tissue and deep muscle using microwatt laser sources (FDA class 1), while light at higher power levels (FDA class 3) has been shown to penetrate through 7 cm of muscle and neonatal skull/brain [86].

The use of SiO_2_/Au nanoparticles (nanoshells) as NIR-absorbing tags is also considered for the photothermal ablation of solid tumors [88]. A pilot study on patients with refractory head and neck cancer is currently being conducted [89]. Au/AuS sulfide NIR-absorbing nanoparticles (35–55 nm) provide higher absorption than nanoshells (98% absorption and *2%* scattering for Au/AuS *versus* 70% absorption and 30% scattering for SiO_2_/Au nanoshells) as well as potentially better tumor penetration [90]. Other nanoparticles used in NIR include hollow gold nanoparticles, which are smaller than SiO_2_/Au nanoshells thus giving them prolonged blood circulation half-life and increased chances of reaching the tumors [91]. Maltzahn *et al.* [92] demonstrated that (PEG)–protected gold nanorods exhibit superior spectral bandwidth, higher photothermal heat generation per gram of gold and longer circulation half-life when compared to gold nanoshells, as well as an approximately two-fold higher X-ray absorption than a clinical iodine contrast agent. NIR-absorbing nanoparticles have also been functionalized with anti-HER2 antibodies to achieve tumor targeting in medulloblastoma cells [93]. Hollow gold nanoparticles were loaded with an a-melanocyte-stimulating hormone analog [90], a potent agonist of melanocortin type-1 receptor overexpressed in melanoma, demonstrating selective photothermal ablation of B16/F10 melanoma. Nanoshells have been loaded into cells of monocyte lineage, which acted as carriers. Once incorporated into human breast tumors in nude mice, the photoinduced cell death of nanoparticle-loaded macrophages was able to induce the death of malignant cells in the tumor's hypoxic microenvironment [94]. Current studies are focused on engineering more efficient NIR-absorbing nanomaterials and on their functionalization with targeting moieties.

Compared to currently available non-invasive procedures with capabilities of increasing the temperature of target tumors, the main drawbacks of magnetic and NIR-absorbing nanoparticles arise from their necessarily invasive nature as well as from the relatively indiscriminate nature of the tissue damage. Due to their efficient intracellular uptake, concerns regarding acute and long-term effects of inorganic nanoparticles accumulation and cytotoxicity are emerging in the biomedical research community [95-97]. Despite the increasing number of newly developed nanoparticles designed for hyperthermia applications, the number of studies addressing their toxicity is low [98]. Collected data indicate that size, crystallinity, shape and surface chemistry strongly influence the mechanism of inorganic nanoparticle internalization by cells, their biodistribution, metabolism and potential toxicity, highlighting the great importance of increasing understanding of healthy and tumor cell interactions with nanoparticles. It is expected that ongoing studies will help reconcile conflicting data and demonstrate the safety of inorganic particles to those reporting transient or acute *in vivo* toxicity.

### 3.4. Gene Therapy

Gene therapy aims to treat diseases by introducing DNA, RNA, small interfering RNA and antisense oligonucleotides into specific target cells or tissues to restore missing functionality and to eradicate pathogenic dysfunction. The therapeutic gene material is delivered to specific target cells using efficient vectors that aim to sustain stable, regulated gene expression without creating unwanted side effects. Viral carriers, organic cationic compounds, recombinant proteins and inorganic nanoparticles are the four kinds of carriers currently being explored for gene delivery applications [99,100]. All of them show advantages and disadvantages, but none of them fulfill the criteria for an ideal vector. Indeed, viruses can be regarded as nanoparticles due to their dimensions, regular geometries and well-characterized surface properties. The most widely used viral vectors for gene transfer include adenoviruses (Ad), which are the dominant gene delivery systems in clinical settings, adeno-associated viruses, herpes simplex-1 viruses, retroviruses and lentiviruses [101]. Viruses are very efficient carriers; however, some of them have limited DNA cargo capacity, can cause immunogenicity and toxicity and their manufacture is rather expensive. In general, synthetic delivery systems prevent specific immune responses and may carry higher amounts of material, without strict limitations on the size of the genetic drugs.

The concept of gene therapy was initially envisioned in the 1970s, but due to the cumbersome nature of the testing required to design and produce effective and safe vectors, gene therapy systems were not fully developed until the early 1980s. The first clinical trials were approved in 1989, and during the 1990s numerous vectors carrying various therapeutic genes were engineered, and their usefulness was tested in preclinical studies. Due to a simplistic belief in the straightforward success of gene therapy, many of these viral vectors rapidly moved to clinical settings. Although success could be demonstrated in some early clinical studies, even when conducted with far from perfect vectors, serious adverse effects and patient deaths led to rigorous regulation of gene therapy protocols for human use. The evolution of currently successful cancer strategies discussed in Sections 1 and 2 also included significant failures and setbacks, which did not restrain investments in chemotherapy and immunological therapies. However, the pharmaceutical industry has not yet developed a single cancer gene therapy product, and so the development of genetic medicines has been left to academic institutions and small biotechnology companies. In addition, the drawbacks of clinical trials for gene therapy led to extended periods of severe cuts in public research funding. The FDA has not yet approved a human gene therapy product for sale, although gene-related research is growing rapidly and many clinical trials are ongoing. Most of these are in Phase I or II and are aimed at dose determination and toxicity assessment [102]. Due to the unknown safety profile of gene vectors, design and approval of human trials were facilitated for life-threatening diseases. Approximately 1,500 trials have been conducted worldwide since 1989, and more than two-thirds of them were conceived for cancer diseases. Due to the complex nature of cancer, the numerous gene therapy approaches for fighting it include strategies for restoring mutant suppressor gene functions, inactivating oncogenes, expressing suicide genes and eliciting protective immune responses [103]. Oncolytic viruses have also been engineered that exploit tumor cells characteristics by replicating them in these target cells as a method for improving the dissemination of biological agents in solid tumors [104]. For the delivery of therapeutic genes encoding proteins with cytotoxic or anti-angiogenic actions, transcriptional targeting using regulatable promoters has been explored as a way of restricting transgene expression to an optimal therapeutic window [105].

To date, there are two gene therapy products available on the market for clinical use, both of which have been approved for cancer treatment in China. Since 2004 China has been the only country in the world where gene therapy is licensed for practice. These products are adenoviral vectors marketed under the brand names Gendicine™ and Oncorine™ [106,107]. Gendicine™ is a p53-overexpressing, replication-incompetent Ad for the treatment of head and neck squamous cell cancer in combination with radiotherapy. Oncorine is an E1B-55K-gene-deleted oncolytic Ad, similar to the discontinued Onyx-015 [107]. A few examples of viruses that have almost reached the market are given below. Cerepro® (sitimagene ceradenovec) is an adenoviral vector containing the herpes simplex virus thymidine kinase gene cDNA under the control of a cytomegalovirus promoter, manufactured by Ark Therapeutics Ltd., for the treatment of high-grade glioma with oral ganciclovir [108]. Cerepro® demonstrated significant efficacy in a recent Phase III trial, but a further trial is still required before approval in order to provide a sufficient level of evidence of clinical benefit [109]. Similar to Gendicine™, Advexin™ (contusugene ladenovec; ING21) was developed by Introgen Therapeutics Inc. as a replication-impaired, adenoviral vector carrying the p53 tumor suppressor gene under the control of a constitutive viral promoter. Numerous human cancers have abnormalities in some of the molecules associated with the p53 pathway, contributing to tumor resistance to a variety of conventional therapeutics. Preclinical data has demonstrated increased amounts of p53 wild-typere protein after transduction with Advexin&trade, and Phase II and III trials were conducted in unresectable recurrent head and neck squamous cell carcinoma [110]. Responders to the adenovirus therapy had a characteristic p53 profile, with either low expression of mutated p53 or wild-type p53 inactivated by upregulation of inhibitors. Genetic immunotherapy was conceived to deliver immune mediators as an efficient and safe approach that also prevents the need to produce and purify large amounts of recombinant proteins [111]. TNFerade™, developed by GenVec [112], is a second-generation adenovirus vector containing E1, E3 and E4 deletions harboring a TNF-α gene, functionally controlled by the radiation-inducible EGR-1 promoter. TNFerade™, was successfully tested in multicenter Phase II and III randomized controlled trials in combination with chemoradiation in patients with locally advanced pancreatic cancer [113]. Despite initially encouraging results, GenVec stopped the phase III trial in March 2010, as an interim analysis could not demonstrate relevant evidence of effectiveness. An example of a retroviral vector in cancer gene therapy is Rexin-G®, currently in clinical trials for advanced pancreatic, metastatic breast cancer, osteosarcoma and soft tissue sarcoma [114]. Rexin-G® is a replication-incompetent, collagen-targeted vector, encoding a dominant negative mutant of the human cyclin G1 gene, which makes it lethal to cancer cells [115,116]. Impressive results were obtained in Phase I and II clinical trials, which demonstrated unprecedented tumor control, prolonged survival and clinical remissions in late-stage cancer patients [117].

Genomic and proteomic technologies are quickly evolving to detect specific molecular targets in patient tumor samples, fulfilling the promise of a personalized treatment approach. Information collected from these emerging technologies will help engineer vectors that carry therapeutic genes specifically targeted to the specificities of individual tumor properties. It is now envisioned that future cancer gene therapies will use a combination of viral and non-viral vectors tailored to meet patient-specific tumor characteristics. Consequently, many research groups have focused their efforts on the generation of synthetic carriers that incorporate features that mimic the biological mechanisms of viral gene delivery. The ideal synthetic vector would incorporate a polycationic sequence to condense nucleic acids and a coating to evade the reticuloendothelial system. It would exhibit colloidal stabilization properties to prevent accumulation in the lung capillaries, and would contain specific target-cell entry, endosomal escape and nuclear localization signals. The goal is to synthetically manufacture biodegradable vectors than can be administered systemically to reach micro metastases. These carriers were initially prepared from polymers, lipids and dendrimers [118]. The first non-viral gene therapy trial was conducted in 1991, on patients with advanced melanoma who received intratumor injection of DNA-liposome complexes [119]. The results demonstrated for the first time the safety and feasibility of cancer treatment by gene therapy protocols using non-biological carriers. Cationic polymers have demonstrated superior gene transfer properties to those of polymers having anionic or neutral charge at physiological pH. However, most clinical trials have been conducted with carriers classified as safe [120], such as the nonamine polymers polyvinyl pyrrolidone and poly(lacid co-glycolic acid). Allovectin-7™, a registered trademark of Vical Incorporated (San Diego, CA, USA) is a promising cancer gene therapy product formulated with a cationic lipid system. Allovectin-7™ contains a bicistronic plasmid encoding human leukocyte antigen-B7 and beta-2 microglobulin. This plasmid allows the immune system to recognize metastatic melanoma lesions as foreign by incorporating a MHC class I complex into the tumor through direct injection, as demonstrated in Phase I/II trials [121]. A Phase III trial is currently being conducted to compare the efficacy of Allovectin-7™ to conventional chemotherapy. Encouraging results were also obtained in a recent Phase I trial conducted on women with recurrent, chemotherapy-resistant ovarian cancer to assess the safety and tolerability of a plasmid carrying the human gene for interleukin-12 plasmid formulated with a synthetic lipopolymer, polyethylene glycol-polyethyleneimine-cholesterol [122]. Currently, numerous nanostructured systems are being developed and tested in preclinical studies. For example, self-assembled nanoparticles containing siRNA, carrier DNA, protamine and lipids, including polyethylene glycol and a ligand, anisamide, to target cancer cells were prepared and tested by Li *et al.* [123]. These authors demonstrated the high efficiency of these systems in delivering genetic material to xenograft tumors after intravenous administration in athymic nude mice. Folate groups have also been linked to liposomes for siRNA delivery, which resulted in significant suppression of xenograft growth in mice [124]. Folate-PEG-polymeric nanoparticles have also been tested *in vivo* for suicide gene therapy applications, using ganciclovir as a prodrug [125]. PEG-modified gelatin-based nanocarriers have been used *in vivo* to deliver plasmid DNA encoding for the soluble form of the extracellular domain of the vascular endothelial growth factor receptor-1 (VEGF-R1 or sFlt-1) in anti-angiogenic therapy [126]. Upon intravenous administration, overexpressed sFlt-1 was therapeutically active as shown by suppression of the xenograft tumor growth. Nanoparticles also offer the ability to monitor the delivery of genetic material. Tan *et al.* [127] were able to synthesize chitosan-based nanoparticles encapsulating quantum dots coupled to siRNA and demonstrate efficient silencing and transfection tracking. Finally, inorganic nanoparticles are also under development, which, despite their low synthesis efficiency, have the significant advantage of low toxicity and easy functionalization [100]. For example, magnetic liposomes have also been tested in magnetic hyperthermia settings to induce therapeutic TNF-α expression driven by the promoter of the stress-inducible gadd153 gene [128]. The combined thermal and gene therapy treatment significantly arrested tumor growth in nude mice, which encouraged the refinement of this type of cancer gene therapy, which was then successfully tested in preclinical studies [129].

After more than two decades of cancer gene therapy using biological vectors, preclinical studies yielded excellent results and clinical trials reported satisfactory results in terms of reporting mild or no long-term toxicity. However, a real breakthrough cannot be claimed in clinical therapy. The reasons for the different outcomes of preclinical and clinical trials include the inherent limitations of rodent models, which develop homogeneous tumors arising from clonal cell lines, while tumors found in clinical practice are composed of heterogeneous cell types. The therapies described in Sections 1 and 2 also confronted similar limitations during their development. The main players in gene therapy, vectors and transgenes, will evolve to achieve the highest possible degree of specificity for targeting cancer cells. Nanotechnology has already engineered powerful non-biological carriers of a variety of therapeutic genes that have demonstrated efficacy and safety in preclinical tests. Since current knowledge of cancer cell biology is far from complete, ongoing and future clinical trials with these synthetic systems are expected to suffer similar drawbacks in terms of efficacy as those experienced with viral gene therapy systems. As we have seen from other therapies that have already been incorporated into the clinical routine of cancer treatment, the success of cancer gene therapies will be preceded by many failures, which will likely be due to a greater extent to our technological limitations than to flaws in their general concept.

## 4. Conclusions

This review has tried to summarize the history and evolution of the most common types of cancer treatments available today, but also new therapies under study in the last years. In addition to surgery, chemotherapy, radiation therapy, hyperthermia, photodynamic therapy or immunotherapy, new therapies are now at different stages of development trying to decrease drug toxicity in health tissues and increase efficacy by targeting tumor angiogenesis, by exploring cell and gene therapy, or by using new nanostructures for diagnosis or therapeutic purposes. Nanotechnology is offering new products, which either used alone, due to their intrinsic properties, or in combination with other biomolecules (anti-tumoral drugs, folic acid, albumin, antibodies, aptamers) could be used to target cancer cells.

However, the history tells us that the fight against cancer is not an easy task. Many types of cancers are able to resist to conventional therapies, and different combinations of drugs and therapies (e.g., surgery together with radiotherapy and chemotherapy) are usually the only way to destroy tumoral cells. This may be also true for the new therapies arriving now to the clinic. Much more studies are required but these new ways of treatment are opening doors to hope for many patients waiting for a successful therapy.

## Figures and Tables

**Table 1.** List of monoclonal antibodies, including the target antigen, therapeutic or diagnostic indication, source and data of approval by the agencies.

**Antibody**	**Company** *	**Target**	**Indication**	**Source**	**Approval** *
3F8	Memorial Sloan-Kettering Merck	GD2	Detection and treatmenf of neuroblastoma	MOUSE; also with ^124^I or ^131^I	Clinical trials
Abciximab **(ReoPro®)**	Centocor B.V. Eli Lilly& Co.	Platelet glycoprotein GPIIb/IIIa.	High risk angioplasty	CHIMERIC Fab fragment	**FDA 1994**
Abagovomab	Menarini	Act as surrogate antigen	Ovarian cancer	MOUSE anti-idiotype	Clinical trials
Adalimumab **(Humira ® Trudexa®)**	Abbott Laboratories	TNFα	Autoinmune disorders like arthritis reumatoid, psoriasis, Crohn's disease	HUMAN	**FDA 2002 EMEA 2003**
Adecatumumab (MT201)	Micromet (MITI)	EpCAM-CD326	Tumor cells (prostate, breast cancers)	HUMAN	Clinical trials
Afelimomab**(Segard** ™)	BASF, Abbot laboratories	TNFα	Sepsis	MOUSE (Fab')2 fragment	Failure of clinical trials
Afutuzumab (Obinutuzumab) (GA-101, RO5072759)	Hoffmann-La Roche	CD20	Lymphoma	HUMANIZED	Clinical trials
Alacizumab pegol (CDP791, gl65 DFM-PEG)	Celltech, UCB	VEGFR2	Non-small cell lung cancer	HUMANIZED F(ab')2 fragment- pegylated	Phase II Clinical trials
ALD518 (BMS-945429)	Alder Biopharmaceuticals, Inc. / Bristol-Myers Squibb	IL-6	Rheumatoid arthritis	HUMANIZED aglycosylated	Phase II Clinical trials
Alemtuzumab **(Campath-1H®; MabCampath®)**	Genzyme	CD52	Chronic lymphocytic leukaemia; T cell lymphoma	HUMANIZED	**FDA 2001 EMEA 2001**
Altumomab pentetate **(Hybri-CEAker™)**	Hybritech incorporated	CEA	Diagnosis of colorectal cancer	MOUSE- pentetate-^l11^In	**FDA orphan product 1990**
Anatumomab mafenatox **(ABR-214936)**	Active Biotech	Glycoprotein 5T4	Non-small cell lung cancer	MOUSE Fab fragment-superantigen *staphylococcal enterotoxin E*	Phase II Clinical trials
Anrukinzumab (IMA-638)	Wyeth Pharmaceuticals	IL-13	Asthma, colitis ulcerosa	HUMANIZED	Clinical trials
Apolizumab (HulDIO, **REMITOGEN™ SMART™)**	PDL (Protein Design Labs) BioPharma	HLA-DR β	Non-Hodgkin lymphoma, Chronic lymphocytic leukemia	HUMANIZED	Phase II Clinical trials
Arcitumomab (CEA-Scan®)	Immunomedics Inc.	Carcinoembrionic antigen	Detection of tumors	MOUSE Ig Fragment- ^99m^_Tc_	**FDA 1996 EMEA1996 Retired 2005**
Aselizumab	?	CD62L	Immunosuppressive drug	HUMANIZED	Clinical trials
Atlizumab (Tocilizumab) **(Actemra®, RoActemra®)**	Hoffman-la Roche Chugai Pharmaceuticals	IL-6 receptor	Rheumatoid arthritis	HUMANIZED	**EMEA 2009 FDA 2010**
Atorolimumab	?	Rhesus factor	Immunosuppresive drug	HUMAN	?
Bapineuzumab	Wyeth / Elan / Pfizer / J&J	β amyloid plaques	Alzheimer's disease/glaucoma	HUMANIZED	Clinical trials
Basiliximab **(Simulect ®)**	Novartis Phamaceutical Corp.	CD25	Prevent rejection in organ transplantation	CHIMERIC	**FDA 1998 EMEA 1998**
Bavituximab	Peregrine Pharmaceuticals, Inc	Phosphatidylserine	Cancer, viral infections	CHIMERIC	Clinical trials
Bectumomab **(LymphoScan®)**	Immunomedics, Inc	CD22	Non-Hodgkin lymphoma	MOUSE Fab'fragment- ^99m^_Tc_	?
Belimumab (LymphoStat-B) **(Benlysta®)**	Human Genome Sciences GSK	BAFF (B cell activation factor)	systemic lupus erythematosus	HUMAN	**FDA 2011**
Benralizumab (BIW-8405, MEDI-563)	Medlmmune Inc.	CD125 (IL-5 receptor)	Asthma	HUMANIZED	Phase II Clinical trials
Bertilimumab (CAT-213)	Cambridge Antibody Technology; Ico Therapeutics	CC111 (Eotaxinl)	Severe allergic disorders	HUMAN	Clinical trials
Besilesomab **(Scintimun®)**	Bayer Schering Pharma A. / CIS bio international	Carcinoembrionic antigen	Metastasis and inflammatory lesions	MOUSE- ^99m^Tc	**EMEA 2009**
Bevacizumab **(Avastin®)**	Genentech Inc./ Roche	VEGF-A	Cancer, age related macular degeneration	HUMANIZED	**FDA 2004 EMEA 2005**
Biciromab(^l11^In) **(FibriScint™)**	Centocor	Fibrin II, β chain	Thromboembolism diagnosis	MOUSE Fab'fragment-^111^In	Withdrawn during clinical trials
Bivatuzumab mertansine	Boehringer Ingelheim	CD44 v6	Squamous cell carcinoma	HUMANIZED-mertansine	Clinical trials
Blinatumomab	Micromet Inc, Medlmmune	CD19/CD3	Non-Hodgkin lymphoma, acute lymphoblastic leukemia	MOUSE	**Orphan drug (EME A/FDA)**
Brentuximab vedotin (SGN-35 and previously cAOO-vcMMAE)	Seattle Genetics and Millennium	CD30	Anaplastic large cell lymphoma; Hodgkin lymphoma	CHIMERIC (mouse/human)-auristatine	Clinical trials
Briakinumab (ABT-874)	Cambridge Antibody Technology; Abbott Laboratories	IL-12andIL-23	Psoriasis, rheumatoid arthritis, inflammatory bowel disease, and multiple sclerosis.	HUMAN	Clinical trials Ozespa, withdrawn for EMA
Canakinumab **(Ilaris®)**	Novartis Phamaceutical Corp.	IL-1β	Ceryopyrin-associated periodic syndromes	HUMAN	**FDA 2009 EMEA 2009**
Cantuzumab mertansine (huC242-DMl, SB408075)	GlaxoSmithKline / ImmunoGen Inc.	Mucin CanAg	Colorectal tumor, Pancreatic cancers	HUMANIZED-mertansine	Phase I/II Clinical trials
Capromab Pendetide (^111^In) **(ProstaScint®)**	Cytogen Corp.	Prostate antigen	Detection of prostate tumor.	MOUSE-pentetide-^111^In	**FDA 1996**
Catumaxomab **(Removab®)**	TRION Pharma	EpCAM and T cells-	Malignant ascitis with EpCAM-positive carcinomas	trifuncional antibody	**EMEA 2009**
**^125^I**-CC49	**?**	TAG-72	Detection of tumors	MOUSE-^125^I	no tumour response, in Phase I and II trials
Cedelizumab (ORTHOCLONE OKT4 A)	Centocor Ortho Biotech Products LP	CD4	Prevention of organ transplant rejections and the treatment of autoimmune diseases	HUMANIZED	Phase II Clinical trials
Certolizumab pegol **(Cimzia®)**	UCB	TNFα	Morbus Crohn, rheumatoid arthritis	HUMANIZED Fab-pegylated	**FDA 2008**
Cetuximab, C-225 **(Erbitux®)**	Imclone Systems/ Bristol-Myers Squibb/ Merck KgaA	EGFR	Colorectal, Head and neck cancer	CHIMERIC	**FDA 2004 EMEA 2004**
ch-TNT	Shanghai Medipharm Biotech	DNA associated antigens	Advanced lung cancer	CHIMERIC-^131^I	**China 2003**
Citatuzumab bogatox VB6-845		TACSTD1	Ovarian cancer, solid tumors	HUMANIZED Fab fragment-bouganin	Pre-clinical trials
Cixutumumab	Imclone Systems	IGF-1 receptor	Solid tumors	HUMAN	Clinical trials
Clenoliximab		CD4	Rheumatoid arthritis	CHIMERIC (primate/human)	
Clivatuzumab tetraxetan yttrium (90 Y)	Immunomedics Inc.	MUC1	Pancreatic cancer	HUMANIZED	Phase II Clinical trials
Conatumumab (AMG-655)	Amgen	TNFRSF10B, TRAIL-R2 (CD262)	Solid tumors	HUMAN	Clinical trials
CR6261	The Scripps Research Institute, Crucell	Influenza A hemagglutinin	Influenza virus infection	HUMAN	Preclinical trials
Dacetuzumab (SGN 40 or huS2C6)	Seattle Genetics	CD40	Non- Hodgkin's lymphoma and hematological malignancies	HUMANIZED	Clinical trials
Daclizumab (Zenapax®)	Hoffman-La Roche	CD25 (IL-2 receptor)	Refractory unstable angina. Allograft rejection	HUMANIZED	**FDA 1997 EMEA 1999**
Daratumumab	Genmab	CD38	Multiple myeloma	HUMAN	Clinical trials
Denosumab XGEVA **(Prolia®)**	XGEVA, Amgen	RANKL	Postmenopausal osteoporosis	HUMAN	**FDA 2010 EMEA 2010**
Detumomab	**?**	B cell lymphoma	B cell lymphoma	MOUSE	?
Ecromeximab (KW2871)	Kyowa Hakko Kogyo Co., Ludwig Institute for Cancer Research	GD3 ganglioside	melanoma	CHIMERIC (mouse/human)	Phase I/II Clinical trials
Eculizumab **(Soliris®)**	Alexion Pharmaceuticals	C5 Complement factor	paroxysmal nocturnal hemoglobinuria	HUMANIZED	**FDA 2007 EMEA 2007**
Edobacomab (E5, XMMEN-0E5)	Pfizer	Endotoxin	Sepsis	MOUSE	Clinical trials
Edrecolomab **(Panorex®)**	Imaging Sciences L1c	EpCAM (17-1A)	Colorectal cancers	CHIMERIC	**German approval 1995**
Efalizumab **(Raptiva®)**	Genentech Inc./ Roche	CDlla	Psoriais	HUMANIZED	**FDA 2003. Recommended suspension. Withdrawn from market 2009**
Efungumab (Mycograb®)	NeuTec Pharma (Novartis)	Fungal HSP90	Candida infection	HUMAN single chain variable fragment (scFv)	Discontinued the development on 2010
Elotuzumab (HuLuc63)	PDL BioPharma Bristol Myers	SLAMf7(CD319)	Multiple myeloma	HUMANIZED	Clinical trials
Elsilimomab (B-E8)	**?**	IL-6	Lymphoma/Myeloma	MOUSE	preliminary Clinical trials
Enlimomab pegol	Boehringer Ingelheim Pharmaceuticals	ICAM-1 (CD54)	Immunomodulator, renal transplant rejection	MOUSE	Clinical trials
Epitumomab cituxetan ^90^Y	**?**	Episialin	Several type of cancers. Epithelial Ovarian cancers	MOUSE- ^90^Y	Phase II
Epratuzumab	UCB and Immunomedics	CD22	Autoinmune disorders such as lupus, Cancer	HUMANIZED	Clinical trials
Erlizumab (rhuMAbCD18)	Genentech/Roche	CD18	Heart attack, stroke, traumatic shock	HUMANIZED F(ab')_2_ fragment	Dropped
Ertumaxomab **(Rexomun®)**	Fresenius Biotech GmbH / TRION Pharma	HER2/neu, CD3	Breast cancer	RAT/MOUSE HYBRID trifucntional antibody	Phase II Clinical trials
Etaracizumab o Etaratuzumab MEDI-522 **(Abegrin® or Vitaxin)**	Medlmmime, Inc.	Integrin αv *β*3	Several type of cancers	HUMANIZED	Clinical trials
Exbivirumab	**?**	Hepatitis B surface antigen	Hepatitis B infections	HUMAN	Preclinical
Fanolesomab **(NeutroSpec™)**	Palatin Technologies	CD15	Apendicitis	MOUSE IgM-^99m^Tc-	**FDA 2004 Suspended in 2005**
Faralimomab	**?**	Interferon receptor	Immunomodulator	MOUSE	**?**
Farletuzumab (MORAb-003)	Morphotek	FR-α	Ovarian cancer	HUMANIZED	Phase III Clinical trials
Felvizumab (SB 209763)	Centocor Inc. / GlaxoSmithKline	Respiratory syncytial virus	Infection by RSV	HUMANIZED	Phase III Clinical trials
Fezakinumab (ILV-094)	Wyeth - Pfizer	IL-22	Rheumatoid arthritis, psoriasis	HUMAN	Phase II Clinical trials
Figitumumab (CP-751871)	Pfizer	IGF-1 receptor	Various types of cancers	HUMAN	Clinical trials
Fontolizumab (HuZAF_-_™)	Novartis Pharmaceuticals Corp. / PDL (Protein Design Labs) BioPharma	Interferon γ	Auto-immune diseases like Crohn's disease	HUMANIZED	Rheumatoid Arthritis dropped, Phase II Clinical trials
Foravirumab (CR4098)	Crucell	Rabies virus glycoprotein	Infection by rabies virus	HUMAN	Phase II Clinical trials
Fresolimumab	Genzyme	TGF β	Pulmonar fibrosis/cancer	HUMAN	Clinical trials 2009
Galiximab (IDEC-114)	Biogen Idee	CD80	B cell lymphoma, Non-Hodgkin's lymphoma, Psoriasis	CHIMERIC (primate/human)	Phase I/II Clinical trials
Gantenerumab (R1450)	F. Hoffmann-La Roche Ltd.	β amyloid	Alzheimer's disease	HUMAN	Phase I Clinical trials
Gavilimomab (ABX-CBL)	Abgenix	CD 147	Graft *versus* host disease	MOUSE	Phase II/III Clinical trials
Gemtuzumab ozogamicin **(Mylotarg®,**CMA-676)	Wyeth → Pfizer	CD33	Relapsed acute myeloid leukaemia	HUMANIZED-Calicheamicin	**FDA 2000 Suspended in US on 2010**
Girentuximab **(Rencarex®** cG250, WX-G250)	Wilex AG, Ludwig Institute for Cancer Research	Carbonic anhydrase 9 (CA-LX, MN, G250)	Renal carcinoma	CHIMERIC	Phase III Clinical trials
Girentuximab **(Redectane®,** 124I_cG250, 1241 WX-G250)	Wilex AG, Ludwig Institute for Cancer Research	Carbonic anhydrase 9 (CA-IX, MN, G250)	Renal mass, kidney tumors	CHIMERIC	Phase III Clinical trials
Glembatumumab vedotin(CR011,CDX-011)	Celldex Therapeutics, Inc.	GPNMB (transmembrane glycoprotein NMB)	Cancer cells expresing NMB: melanoma, breast cancer	HUMAN- Auristatin	Phase II Clinical trials
Golimumab **(Simponi®)**	J&J	TNFα	Rheumatoid arthritis, psoriatic arthritis and ankylosing spondulitis	HUMAN	**FDA 2009 EMEA 2009**
Gomiliximab	IDEC Pharmaceuticals Corporation	CD23	Allergic asthma	CHIMERIC (primate/human)	withdrawn
Ibalizumab (TMB-355)	Tanox; TaiMed Biologies	CD4	HIV entry inhibitor	HUMANIZED	Clinical trials
Ibritumomab tiuxetan **(Zevalin®)**	Biogen IDEC Pharmaceuticals Corp.	CD20	Non-Hodgkin lymphoma	MOUSE Ig- ^90^Y	**FDA 2002 EMEA 2004**
Igovomab **(Indimacis-125®)**	CIS Bio international	MUC16CA-125	Ovarian cancer	MOUSE conjugated to ^111^In	**FDA 1996, EC withdrawal 1999**
Imciromab-Pentetate **(Myoscint™**)	Centocor	Heart myosin	Detection of heart disease	MOUSE conjugated to ^111^In	**FDA Orphan product 1989; Withdrawn in 1993**
Infliximab **(Remicade®)**	Centocor (J&J)	TNFα	Psoriasis, Crohn's disease, ankylosing spondylitis, psoriatic arthritis, rheumatoid arthritis and ulcerative colitis.	CHIMERIC (mouse/human)	**FDA 1998 /EMEA 1999**
Inolimomab	OPI (Orphan Pharma International)	IL2RA, CD25	Graft-veraMi-host disease	MOUSE	Phase II/III Clinical trials
Inotuzumab ozogamicin (CMC-544)	Wyeth - Pfizer	CD22	Diffuse large B cell lymphoma, Non-Hodgkin lymphoma	HUMANIZED -Calicheamicin	Phase II Clinical trials
Ipilimumab (MDX-101) **(Yervoy™)**	Bristol-Myers Squibb.	CD 152 (CTLA-4)	Activator of the immune system: late stage melanoma and other type of tumors	HUMAN	**FDA 2011**
Iratumumab (MDX-060)	Medarex, Inc.- Bristol-Myers Squibb	CD30	CD30-positive lymphoma including Hodgkin's disease	HUMAN	Phase II Clinical trials
Keliximab(IDECCE9.1/SB-210396)	Biogen IDEC Pharmaceuticals, SKB	CD4	Immunosuppressor. Severe chronicAsthma, Rheumatoid arthritis	CHIMERIC (primate/human)	Phase III Clinical trials suspended
Labetuzumab (hMN14, **CEACIDE™)**	Immunomedics, Inc	CEA	Colorectal tumor	HUMANIZED	Phase I/II Clinical trials
Lebrikizumab (MILR1444A)	Roche-Genentech	IL-13	Asthma	HUMANIZED	Phase II Clinical trials
Lemalesomab	?	NCA-90 (granulocyte antigen)	Diagnosis of inflammatory lesions	MOUSE	?
Lerdelimumab (CAT-152)	Cambridge Antibody Technology	TGF β	Immunosuppresor. Glaucoma	HUMAN	Phase III Clinical trials
Lexatumumab (ETR2-ST01)	HGS; Cambridge Antibody Technology	TRAIL-R2 (AP02)	Tumors	HUMAN	Clinical trials
Libivirumab	?	Hepatitis B surface antigen	Hepatitis B infection	HUMAN	Preclinical
Lintuzumab	Seattle Genetics	CD33	acute myeloid leukemia	HUMANIZED	Clinical trials
Lorvotuzumab mertansine IMGN901	ImmunoGen, Inc	CD56	Small cell lung cancer, ovarian cancer	HUMANIZED -mertansine	Orphan drug; clinical trials
Lucatumumab	Novartis Pharmaceuticals Corp	CD40	Cancer like multiple myeloma, non-Hodgkin's or Hodgkin's lymphoma	HUMAN	Clinical trials
Lumiliximab(IDEC-152,P5E8)	Biogen IDEC Pharmaceutical	CD23	Chronic lymphocytic leukaemia, Allergic asthma	CHIMERIC (primate/human)	Phase I/II Clinical trials
Mapatumumab	Cambridge Antibody Technology and Human Genome Sciences, Inc.	TRAIL-receptor (death receptor 4	Several tumors	HUMAN	Clinical trials
Maslimomab	?	T cell receptor	Immunosuppresor	MOUSE	?
Matuzumab (EMD 72000)	Merck Serono; Takeda Pharmaceutical,	EGFR	Several tumors	HUMANIZED	Dropped
Mepolizumab	GlaxoSmithKline	IL-5	Hypereosinophilic syndrome	HUMANIZED	Clinical trials
Metelimumab (CAT-192)	Cambridge Antibody Technology	TGF β1	Scleroderma	HUMAN	Dropped
Milatuzumab	Immimomedics, Inc	CD74	Multiple myeloma	HUMANIZED-doxorubicin	Clinical trials
Minretumomab	?	TAG-72	Cancer	MOUSE	?
Mitumomab (BEC2)	ImClone Systems Inc./ Memorial Sloan-Kettering Cancer Center/Merck KgaA	GD3 ganglioside	Melanoma and Small cell lung carcinoma	MOUSE	Phase III Clinical trials
Morolimumab	?	Rhesus factor	Immunosuppresor	HUMAN	?
Motavizumab (Numax)	Medlmmune	RSV glycoprotein F	Prevention of respiratory syncitial inf.	HUMANIZED	**FDA withdrawn 2010**
Muromonab-CD3. **(Orthoclone OKT3™)**	Ortho Biotech, Inc. (subsidiary of J&J) Janssen-Cilag	CD3	Prevention of organ transplant rejection	MOUSE	**FDA 1986 EMEA 1987**
Nacolomab tafenatox	?	C242	Colorectal tumor	MOUSE-enterotoxin A from Staphylococcus aureus	?
Naptumomab estafenatox (ABR-217620, ANYARA, TTS CD3)	Active Biotech AB	TPBG (trophoblast glycoprotein, 5T4)	Several tumors	MOUSE Fab fragment-enterotoxin E from Staphylococcus aureus	Clinical trials
Natalizumab (Tysabri®)	Biogen Idee and Elan Corp.	Integrin α4 subunit ofa4β1	Multiple Sclerosis, Chron's disease	HUMANIZED	**FDA 2004/ withdrawn/ back on2006/ EMEA only for restricted cases**
Nebacumab (centoxin, HA-1A. septomonab)	Centocor	Endotoxin	Sepsis	HUMAN	Withdrawn in 1993
Necitumumab (IMC-11F8)	ImClone Systems Inc.	EGFR	Several tumors	HUMAN	Clinical trials
Nerelimomab	?	TNFα	TNF inhibitor	MOUSE	**?**
Nimotuzumab **(BIOMab EGFR®) (TheraCIM) (TheraLoc) (CIMAher)**	CIM, Cuba YM Biosciences, Out-licensed to other companies Daiichi Sankyo, Inc (ONLY JAPAN)	EGFR	Squamous cell carcinoma and glioma	HUMANIZED	**Orphan drug FDA, EMEA 2004, Several countries 2005 China; 2006 India.**
Nofetumomab merpentan **(Verluma®)**	Boehringer Ingelheim Pharma KG	Glycoprotein 40 kD	Detection of tumors	MOUSE Fab IgG_2b_-merpentan-^99m^Tc	**FDA 1996**
Ocrelizumab	Hoffman-La Roche Inc.	CD20	Immunosuppresive drug	HUMANIZED	Clinical trials
Odulimomab. (afolimomab. **ANTILFA®)**	Pasteur-Merieux	integrin α L subunit -CDlla	Allograft Transplant rejection	MOUSE	Phase III, not renewed
Ofatumumab **(Arzerra HuMax-CD20®)**	Genmab	CD20	Chronic lymphocytic leukemia	HUMAN	**FDA 2009 EMEA 2010**
Olaratumab (IMC-3G3)	Imclone	PDGF-Rα	Solid tumors	HUMAN	Phase I Clinical trials
Omalizumab **(Xolair®)**	Genentech Inc./ Roche/ Tanox, Inc., Novartis Pharmaceuticals	IgE	Severe asthma.	HUMANIZED	**FDA 2003 EMEA 2005**
Oportuzumab monatox. **(PROXINIUM™ VICINIUM™)**	Viventia Biotechnologies Inc.	EpCAM, and others	Several tumors	HUMANIZED (sc Fv)- Pseudomonas aeruginosa exotoxin A	Phase II/III Clinical trials
Oregovomab **(OVAREX®)**	AltaRex Corp	MUC16, CA-125	Ovarian tumors	MOUSE	Phase II Clinical trials
Otelixizumab TRX4	Tolerx, Inc. AND GlaxoSmithKline. Manufact. by Abbott Laboratories	CD3ε	Type 1 diabetes and other autoimmune diseases	CHIMERIC/HUMANIZED	Clinical trials. Orfan drug status FDA
Pagibaximab	Biosynexus, Glaxo Smith Kline	Staphylococcal lipoteichoic acid	Prevention of sepsis by staphylococcus	CHIMERIC (mouse/human)	**Orphan drug status EMEA 2010**
Palivizumab (Synagis™)	Medimmune Inc.	An epitope of the RSV F protein	Respiratory syncitial virus infection	HUMANIZED	**FDA 1998 EMEA 1999**
Panitumumab (ABX-EGF) **(Vectibix™)**	Amgen/Abgenix	Epidermal growth factor receptor (EGFR)	Metastatic colorectal carcinoma	HUMAN	**FDA 2006 EMEA 2007**
Panobacumab (o Aerumab 11) (KBPA-101)	Kenta Biotech Ltd	Pseudomona aeruginosa serotype ATS 01l	Infection by pseudomona	HUMAN	Phase I/II Clinical trials
Pascolizumab	Centocor Inc. / GlaxoSmithKline	IL-4	Allergy, Asthma	HUMANIZED	Phase II Clinical trials
Pemtumomab **(Theragyn)**	Antisoma pic Abbot Laboratories	MUC1	Ovarian and peritoneal cancer	MOUSE	Clinical trials; orphan drug status in FDA and EMEA
Pertuzumab **(Omnitarg™)**	Genentech	HER2	Tumors	HUMANIZED	Clinical trials
Pexelizumab	Procter & Gamble (P&G) Alexion Pharmaceuticals	C5 Complement component	Reduce side effects of coronary artery bypass grafting and angioplasty	HUMANIZED	Disappointing results on phase III.
Pintumomab technetium -^99m^Tc	?	Adenocarcinoma antigen	Imaging of adenocarcinoma	MOUSE-^99m^Tc	**?**
Priliximab	Centocor	CD4	Crohn's disease and multiple sclerosis	CHIMERIC (mouse/human)	pending
Pritumumab	Nascent Biologies, Inc.	Vimentin	Brain cancer	HUMAN	Clinical trials
Pro 140	Progenies Pharmaceuticals	CCR5	HIV infection	HUMANIZED	Clinical trials/ fast track approval
Rafivirumab (CR57)	Crucell	Rabies virus glycoprotein	Rabies profilaxis	HUMAN	Phase II Clinical trials
Ramucirumab (IMC-1121B)	Pfizer/ImClone Systems Inc	VEGFR-2	Several tumors	HUMAN	Clinical trials.
Ranibizumab (Lucentis®)	Genentech Inc. (Roche) / Novartis	Vascular endothelial growth factor A (VEGF-A)	Wet Macular degeneration	HUMANIZED Fab	**FDA 2006 EMEA 2007**
Raxibacumab (ABthrax)	Human Genome Sciences	Protective antigen of anthrax toxin	Antrax toxin	HUMAN	Phase III Clinical trials
Regavirumab	Teijin	Cytomegalovirus glycoprotein B	Cytomegalovirus infection	HUMAN	Phase I Clinical trials
Reslizumab	Ception Therapeutics, Inc	IL-5	Eosinophil-meditated inflammations	HUMANIZED (from rat)	Phase II Clinical trials
Rituximab (Rituxan®) (MabThera®)	Roche / Biogen Idee	CD20	Non-Hodgkin lymphomas, rheumatoid arthritis	CHIMERIC	**FDA 1997 EMEA 1998**
Robatumumab (SCH 717454)	Schering-Plough.	CD221	Colon sarcoma, Blood cancers	HUMAN	Preclinical and Phase II Clinical trials
Rontalizumab	Genentech Inc.	IFNα	Systemic lupus erythematosus	HUMANIZED	Clinical trials.
Rovelizumab (LeukArrest, Hu23FG2)	Icos	CD11,CD18	Immunosuppresive drug	HUMANIZED	**FDA 1998, dropped 2000**
Ruplizumab (Antova™)	Biogen Idee Ma Inc.	CD154	Rheumatic diseases	HUMANIZED	FSA fast tracked
Satumomab pendetide (OncoScint CR103™)	Cytogen Corp. and Cetus Corp, Lonza Biologies	TAG-72	Ovarian and Colorectal Cancer diagnosis	MOUSE-pentetide- ^11l^In	Withdrawn
Sevirumab (MSL-109) **(Protovir™)**	Novartis Pharmaceuticals Corp. / PDL (Protein Design Labs) BioPharma	CMV	Cytomegalovirus infection in AIDS patients	HUMAN	Phase III
Sibrotuzumab	?	FAP	Tumors	HUMANIZED	
Siltuximab	Centocor Inc.	IL-6	Multiple myeloma and other Tumors	CHIMERIC (mouse/human)	Phase II Clinical trials
Siplizumab (MEDI-507)	BioTransplant, Medlmmune Inc.	CD2	Psoriasis and in the prevention of graft-verms - host disease, and Acute kidney transplant rejection	HUMANIZED (from rat)	Phase II Clinical trials
Solanezumab	Eli Lilly	βamyloid	Alzheimer's disease	HUMANIZED	Phase II Clinical trials
Stamulumab (MYO-029)	Cambridge Antibody Technology, Wyeth Pharmaceuticals	Myostatin	Muscular distrophy	HUMAN	Phase I/II Clinical trials
Sulesomab-Technetium **(Leukoscan®)**	Immunomedics Inc, Nycomed GmbH	NCA-90 (granulocyte cell antigen	Detection of inflammation, diagnosing osteomyelitis	MOUSE- ^99m^Tc	**marketed in European Union**
Tacatuzumab tetraxetan-yttrium (^90^Y) (AFP-Cide)	Immunomedics Inc.	α fetoprotein (AFP)	Cancers	HUMANIZED-tetraxetan-^90^y	withdrawn
Tadocizumab (C4Gl,YM-337)	Yamanochi Pharma America, PDL (Protein Design Labs) BioPharma	Integrin αIIbβ3	Percutaneous coronary interventions	HUMANIZED Fab	Phase II Clinical trials
Talizumab (TNX-901)	Tanox, Novartis	Fc region of IgE	Allergy reactions	HUMANIZED	withdrawn
Tanezumab (RN624)	Pfizer	Nerve growth factor (NGF)	Pain treatment	HUMANIZED	Clinical trials
Taplitumomab paptox	?	CD19	Tumors	MOUSE- conjugated with antiviral protein PAP from *Phytolacca americana*	?
Tefibazumab (Aurexis®)	Inhibitex	Clumping factor A	Severe infectious with Staphylococcus a.	HUMANIZED	Clinical trials
Telimomab aritox	?		Immunosuppresive drug	MOUSE (Fab fragment)-ricin protein	1991, phase I, discontinued
Tenatumomab	Sigma-Tau	Tenascin C	Cancer	MOUSE	?
Teneliximab	?	CD40	Immunosuppresive drug	CHIMERIC (mouse/human)	?
Teplizumab MGA031	Eli Lilly	CD3	Diabetes mellitus type 1	HUMANIZED	Phase III disappointed
TGN1412 (CD28-superMAB)	TeGenero Immuno Therapeutics	CD28	B cell chronic lymphocytic leukemia (B-CLL) and rheumatoid arthritis,	HUMANIZED	Catastrophic systemic organ failure 2006 on phase I
Tigatuzumab (CS-1008)	Daiichi Sankyo, Inc	TRAIL-R2 OrDR5	Several tumors (colorectal, pancreas, ovary)	HUMANIZED	phase II Clinical trials
TNX-650	Tanox	IL-13	Refractory Hodgkin's lymphoma	HUMANIZED	Clinical trials
Tocilizumab o atlizumab **(Actemra®)**	Hoffman-la Roche; Chugai Pharmaceuticals.	IL-6 receptor	Rheumatoid arthritis	HUMANIZED	**EMEA 2009 FDA 2010**
Toralizumab (IDEC 131)	IDEC Pharmaceuticals Corporation	CD154 (CD40 ligand)	Immune thrombocytopenic purpura, lupus nephritis, rheumatoid arthritis)	HUMANIZED	Trials halted on Phase II
Tositumomab **(Bexxar®)**	Corixa Corp and GlaxoSmithKline	CD20	Non-Hodgkin follicular lymphoma	Mouse Ig-^131^I	**FDA 2003**
Trastuzumab **(Herceptin®)**	Genentech Inc. (ROCHE)	ErbB2 (HER2/neu)	Breast cancer	HUMANIZED	**FDA 1998 EMEA 2000**
Tremelimumab o Ticilimumab (CP-675,206)	Pfizer	CD 152 (CTLA-4)	Melanoma/small cell lung cancer/prostate cancer	HUMAN	Clinical trials
Tucotuzumab celmoleukin	EMD pharmaceuticals	EpCAM	Several tumors	HUMANIZED- celmoleukin (IL-2)	Clinical trials
Tuvirumab	PDL (Protein Design Labs) BioPharma	Hepatitis B virus	Chronic hepatitis B	HUMAN	Phase I clinical trials in 2001
Urtoxazumab	TMA-15 TEIJIN	E coli Shiga like toxin II B (STEC 0-157 infection)	Diarrhoea by Escherichia coli (serotype 0121)	HUMANIZED	Phase II Clinical trials
Ustekinumab **(Stelara®)**	J&J	P40fromIL-12 and IL-23	Psoriasis, multiple sclerosis	HUMAN	**FDA 2009 EMEA 2008**
Vapaliximab	(HUVAP) Biotie Therapies (VAP-1 fully human) Turku-Finland	Vascular adhesion protein (VAP-1)	Inflammation	CHIMERIC (mouse/human)	Phase I (2002)
Vedolizumab	Millennium Pharmaceuticals	Integrin α4β7	Ulcerative colitis and Crohn's disease	HUMANIZED	Clinical trials
Veltuzumab	Immunomedics, Inc	CD20	Non-Hodking's lymphoma	HUMANIZED	Clinical trials
Vepalimomab (vepalimomabum)	Biotie Therapies (VAP-1 fully human)	Vascular adhesion protein (VAP-1)	Inflammation	MOUSE HUMAN	Phase 2 enabling work underway.(Scifinder)Immunoglobulin M, anti-(human vascular adhesion protein VAP-1)(mouse monoclonal 1B2μchain) disulfide with mouse monoclonal 1B2 light chain, dimer
Visilizumab **(Nubion)**	PDL BioPharma Inc	CD3	Multiple myeloma and diabetes mellitus type 1	HUMANIZED	Clinical trials
Volociximab	PDL BioPharma and Biogen Idee	Integrin α5β1	Solid tumors	CHIMERIC(mouse/human)	Clinical trials
^99m^Tc -Votumumab **(HumaSPECT®)**	KS Biomedix Ltd, Non commercialized	CTAA16.88 cytokeratin polypeptides	Detection of colorectal tumor. Diagnostic imaging	HUMAN- ^99m^Tc	EC withdrawal
Zalutumumab **(HuMax-EGFR)**	GenMab	EGFR	Squamous cell carcinoma resistant to chemotherapy	HUMAN- BSC	Clinical trials
Zanolimumab **(HuMax-CD4)**	GenMab	CD4	Rheumatoid arthritis/psoriasis/melanoma/T cell lymphoma	HUMAN	Clinical trials
Zolimomab aritox	XOMA Corp CD5 Plus; H65-RTA; Orthozyme CD5plus; XZ-CD5; XomaZyme-H65	CD5	Systemic lupus erythematosus / graft *versus* host disease	MOUSE-ricin protein	the studies failed to show positive effects(Immunotoxin)

*Observation: Commercialization by companies and the state of clinical trials can change during time. It has been compiled from various sources.

**Table 2.** Nanoscale or nanostructured-based therapeutic and diagnostic agents currently marketed for the treatment of cancer.

	**Trademark**	**Composition**	**Indication**	**Company**
	**Improve drug solubility and bioavailability**			
**iposomal formulations**	DaimoXome	Daunorubicin liposomal	Advanced HIV-associated Kaposi's sarcoma	Gilead Sciences Inc. (Foster City, CA, USA) (acquired by Diatos S.A.)
	Doxil/Caelyx	Doxorubicin HC1 liposomal	Ovarian cancer, AIDS-related Kaposi sarcoma, and multiple myeloma	Ortho Biotech (Bridgewater, NJ, USA); Schering-Plough (Kenilworth, NJ, USA)
	DepoCyt	Cytarabine liposomal	Lymphomatous meningitis	Pacira Pharmaceuticals Inc. (San Diego, CA, USA)
	Myocet	Doxorubicin liposomal	Advanced breast cancer	Cephalon (Frazer, PA, USA)
	Onco TCS	Vincristine liposomal	Breast cancer, Hodgkin's disease, Kaposi's sarcoma, and testicular cancer	Enzon Pharmaceuticals, (Bridgewater, NJ, USA)
**Polymeric formulations**	Genexol-PM	Paclitaxel loaded polymeric micelles of m-PEG-PLA	Breast, lung and pancreatic cancers	Samyang Genex Corp. (Seoul, Korea)
	Oncaspar	PEGylated asparaginase	Acute lymphoblastic leukemia	Enzon Pharmaceuticals Inc. (Bridgewater, NJ, USA)
	Zinostatin Stimalmer (SMANCS)	Polymer-protein conjugate (Styrene maleic anhydride-neocarzinostatin)	Hepatocellular carcinoma	Astellas Pahrma Inc. (Osaka, Japan)
	Neulasta/PEG filgrastim	Polymer-protein conjugate	Prevention of chemotherapy-associated neutropenia	Amgen (Thousand Oaks, CA, USA)
**Nanoparticulated**	Abraxane	Paclitaxel albumin-stabilized nanoparticle formulation	Metastatic and recurrent breast cancer.	Abraxis BioScience (Schaumburg, IL, USA), AstraZeneca (London, UK)
**formulation**			(The need for premedication for hypersensitivity reactions caused by the solvents used to solubilize the free drug formulation is eliminated)	
	Emend	Capsule containing pellets of nanocrystalline aprepitant	Used to help prevent nausea and vomiting caused by chemotherapy	Merck & Co., Inc. (Whitehouse Station, NJ USA)
**Magnetic hyperthermia**	NanoTherm nanoparticles	Aminosilane coated iron oxide	Recurrent glioblastoma multiforma	Magforce Nanotechnologies AG (Berlin, Germany)
**Photodynamic therapy**	AuroShell nanoparticles	Gold coated silica nanoparticles	Currently conducting a pilot study in patients with refractory head and neck cancers	Nanospectra Biosciences, Inc. (Houston, TX,USA)
**Controlled drug release**	Brachysil	Nano-structured porous silicon encapsulating radioactive phosphorous (^32^P)	Brachytherapy product currently in development for the treatment of solid tumors (clinical trial in pancreatic cancer)	pSivida Corp. (Watertown MA, USA)
	Gliadel	Biodegradable polymeric wafer loaded with carmustine	Treatment of newly-diagnosed high-grade malignant glioma as an adjunct to surgery and radiation	Eisai Inc. (Woodcliff Lake, NJ, USA)
	***In vivo* diagnosis**			
**Contrasts agents for MRI**	Resovist	superparamagnetic iron oxide (SPIO) nanoparticles coated with carboxydextran	Liver lesions	Bayer Schering Pharma AG (Berli, Germany)
	Feridex/Endorem	SPIO nanoparticles coated with dextrane	Liver lesions	Advanced Magnetics (Cambridge, MA, USA), Guerbet S.A. (Roissy, France)
**Monitoring metastatic prostate cancer**	Cell Search	Immunomagnetic (using magnetic nanoparticles) labelling and immunofluorescent identification of tumor cells	Circulating tumor cell (CTC) test is a simple blood-test that captures and assesses CTCs to determine the prognosis of patients with metastatic breast, colorectal or prostate cancer at any time	Veridex LLC (Raritan, NJ, USA)
